# The characteristics of soil microbial co-occurrence networks across a high-latitude forested wetland ecotone in China

**DOI:** 10.3389/fmicb.2023.1160683

**Published:** 2023-03-21

**Authors:** Di Wu, Hui Bai, Caihong Zhao, Mu Peng, Qi Chi, Yaping Dai, Fei Gao, Qiang Zhang, Minmin Huang, Ben Niu

**Affiliations:** ^1^State Key Laboratory of Tree Genetics and Breeding, Northeast Forestry University, Harbin, China; ^2^The Center for Basic Forestry Research, College of Forestry, Northeast Forestry University, Harbin, China; ^3^College of Life Science, Northeast Forestry University, Harbin, China; ^4^Key Laboratory of Fast-Growing Tree Cultivating of Heilongjiang Province, Forestry Science Research Institute of Heilongjiang Province, Harbin, China; ^5^Institute of Economic Forest of Xinjiang Academy of Forestry Sciences, Urumqi, China

**Keywords:** forested wetland, fungal and bacterial community, co-occurrence patterns, seasonal change, vegetation type

## Abstract

To understand the effect of seasonal variations on soil microbial communities in a forested wetland ecotone, here, we investigated the dynamics of the diversities and functions of both soil bacterial and fungal communities inhabiting three wetland types (forested wetland, shrub wetland and herbaceous vegetation wetland) from forest-wetland ecotone of northern Xiaoxing’an Mountains spanning different seasons. β-diversity of soil microbial communities varied significantly among different vegetation types (*Betula platyphylla*–*Larix gmelinii*, *Alnus sibirica*, *Betula ovalifolia*, and *Carex schmidtii* wetlands). We totally detected 34 fungal and 14 bacterial indicator taxa among distinctive groups by using Linear discriminant analysis effect size (LEfSe) analysis, and identified nine network hubs as the most important nodes detected in whole fungi, bacteria, and fungi–bacteria networks. At the vegetation type-level, bacterial and fungal microbiome living in *C. schmidtii* wetland soil possessed fewer positive interactions and lower modularity than those in other types of wetland soil. Furthermore, we also discovered that ectomycorrhizal fungi were dominant in the fungal microbiota existing in forested and shrub wetland soils, whereas arbuscular mycorrhizal fungi were predominated in those residing in herbaceous vegetation wetland soil. The distribution of the predicted bacterial functional enzymes also obviously varied among different vegetation-types. In addition, the correlation analysis further revealed that the key fungal network modules were significantly affected by the contents of total N and soil water-soluble K, whereas most of the bacterial network modules were remarkably positively driven by the contents of total N, soil water-soluble K, Mg and Na. Our study suggested that vegetation type are substantive factors controlling the diversity, composition and functional group of soil microbiomes from forest-wetland ecotone of northern Xiaoxing’an Mountains.

## Introduction

The dynamics of soil microbial communities, an important component of many ecosystems, are often of broad attention in biogeochemical models (Broadbent et al., 2021; Li J. W. et al., 2022). Soil microbes are of great importance for the improvement of plant productivity, organic matter decomposition, and maintenance of nutrient balance in ecosystems, due to their diverse functions (Tang et al., 2011; Delgado-Baquerizo et al., 2016; Jiao et al., 2018; Ding et al., 2020). They also often are considered as indicators of soil quality, because of which are sensitive to the variation of soil physicochemical properties (Wu et al., 2021; Li M. et al., 2022). Besides, soil functional bacteria and fungi under different nutrient levels can accommodate or withstand environmental disturbance and dynamics by establishing co-occurrence interactions, such as mutualism and competition (Banerjee et al., 2016; Chen et al., 2019). Therefore, the composition and diversity of soil microbial communities has an irreplaceable effect on the biodiversity and functional stability of soil ecosystem (Ren et al., 2022).

Forested wetlands, as crucial natural ecosystems, are located at the interface between forest and aquatic ecosystems, which provide diversified ecological niches for many organisms and serve important ecological functions in protecting biodiversity (Boyce et al., 2012; Janse et al., 2019; Ma et al., 2020). Nevertheless, disturbance of vegetation or other environmental factors may trigger shifts in biodiversity patterns or ecosystem states in wetlands (Heffernan, 2008). It has been demonstrated that changes in composition and diversity of soil microbial communities are guided by changes in the diversity and functional types of plants (Schlatter et al., 2015; Delgado-Baquerizo et al., 2017; Ding et al., 2020). Moreover, seasonal variations can also significantly give rise to changes in plant growth and soil nutrients by affecting plant physiological functions and further influencing soil microbial community composition (Siles et al., 2017; Eslaminejad et al., 2020). However, it is less known whether the diversity of soil microbial communities in natural forested wetland ecotones are more dependent on vegetation or seasonal variations.

Xiaoxing’an Mountains is one of the three main mountainous wetland distribution areas (including Changbai and Daxing’an Mountains) in Northeast China, which is of significant research value in the field of wetland at temperate regions (Sui et al., 2009). Many previous studies on wetlands have focused on soil carbon storage and carbon flux in wetlands (Helbig et al., 2017; Aguilos et al., 2020). The co-occurrence network, by elucidating the relationships between pairs of microbial taxa, can allowed a comprehensive interpretation of assembly and functioning of biological communities, which contribute to decipher more ecological patterns on microbial species in diverse ecosystems (Goberna and Verdú, 2022; Xue et al., 2022). Currently, there is still not comprehensive enough awareness about how the structure of soil microbial networks in wetlands changes across continuous vegetation transition, especially in forested wetland ecotones. In this study, four typical natural wetlands (*B*. *platyphylla*–*L*. *gmelinii*, *A*. *sibirica*, *B*. *ovalifolia*, and *C*. *schmidtii* wetlands) in the temperate zone of China were selected, which were distributed successively along the forest–wetland ecotone in the north of Xiaoxing’an Mountains. The primary aims of this study were: (1) to reveal the distribution characteristics of soil bacterial and fungal functional communities along the environmental gradient of the forested wetland ecotone; (2) to elucidate the key taxa in the community assembly and co-occurrence patterns of soil bacterial and fungal communities from different wetlands that withstand seasonal variation; and (3) to recognize the role of soil microbes in the transformation and competition for nutrients in wetland habitats and to predict whether these functional communities serve an important regulatory function during wetland ecotone succession. Therefore, our study will not only provide new insights into the role of the co-occurrence networks of soil microorganisms in response to soil perturbation, but also a preliminary forecast for the potential ecological succession of forested wetland ecotones.

## Materials and methods

### Experimental site and sampling

The study area is located in Wuyiling Wetland Nature Reserve at Xiaoxing’an Mountains of Northeast China (E129°00′–129°30′, N48°33′–48°50′; 350–550 m asl), in a temperate continental monsoon climate zone. The main types of wetlands in this area include forested, shrub, herbaceous, and moss wetlands and shallow water marshes; forested wetlands occupy the largest area. Rainfall mainly occurs in the summer. The average annual precipitation is 584 mm; snow cover depth, 27 cm; temperature, −1.1°C; and the annual accumulated temperature of ≥10°C is 1,700–2,000°C. The frost-free period during the study was approximately 97 days, and the freezing period was 180 days. The zonal soil type is dark brown soil, whereas azonal soils include meadow soil, swamp soil, and peat soil (Cai et al., 2010).

Four vegetation types were selected as experimental sites in the successive ecotones of forested wetlands, including *B*. *platyphylla*–*L*. *gmelinii* wetland (BLW; forested wetland), *A*. *sibirica* wetland (ASW; forested wetland), *B*. *ovalifolia* wetland (BOW; shrub wetland), and *C*. *schmidtii* wetland (CSW; herbaceous vegetation wetland). Three plots (20 m × 20 m) were prepared for each wetland type. Soil samples were collected in winter (December 2015), spring (May 2016), summer (August 2016), and autumn (October 2016) to study the effect of seasonal factors on the characteristics of soil bacterial and fungal communities. The litter layer was removed, and soil samples (10-spot sampling method) from each plot at depths of 0–10 cm were collected and mixed into a single sample. The soil samples from each plot were sieved to remove plant roots, stones, etc. These samples were stored at −80°C until DNA extraction for soil fungal and bacterial community analysis.

### Determination of soil nutrient contents

Total N content was measured using a Kjeldahl apparatus (BUCHI, Ltd., Flawil, Switzerland). Soil water-soluble calcium (Ca), magnesium (Mg), sodium (Na), potassium (K), and available zinc (Zn) was determined using atomic absorption spectrophotometry (AAS; PinAAcle 900 T, PerkinElmer, Inc., MA, United States).

### The amplification of 16S rRNA gene and its sequences and illumina sequencing

Total microbial DNA was extracted from 0.25 g per soil sample using a Power Soil DNA Isolation Kit (MOBIO Laboratories Inc., United States) according to the manufacturer’s protocol. The extracted DNA was stored at −80°C for soil microbial community analysis. The V3-V4 region of the 16S bacterial gene and the fungal ITS region were amplified to analyze the structure and diversity of bacterial and fungal communities, respectively. The V3-V4 region of the bacterial 16S rRNA gene was amplified with the barcoded primer set 338F (5’-ACTCCTACGGGAGGCAGCAG-3′) and 806R (5’-GGAC TACHVGGGTWTCTAAT-3′; Xu et al., 2016) using the following PCR program: 95°C for 3 min; 27 cycles of 95°C for 30 s, 55°C for 30 s, 72°C for 45 s, and extension for 10 min at 72°C. The fungal ITS region was amplified using the barcoded primer set ITS1F (5’-CTTGGTC ATTTAGAGGAAGTAA-3′) and ITS2R (5’-GCTGCGTTCTT CATCGATGC-3′; Adams et al., 2013) using the following PCR program: 95°C for 3 min; 35 cycles of 95°C for 30 s, 55°C for 30 s, 72°C for 45 s, and extension for 10 min at 72°C. Purified PCR products were pooled and sequenced on an Illumina MiSeq platform according to the standard instructions.

### Illumina sequencing and data analysis

Raw bacterial and fungal DNA sequences were extracted, quality screened, and filtered using Trimmomatic (Bolger et al., 2014). Forward and reverse sequences were merged using Flash (version 1.2.11) with previously described methods (Sui et al., 2019). Operational taxonomic units (OTUs) were generated with a 97% similarity cutoff using UPARSE (version 7.1), and chimeras were removed using the UCHIME algorithm. Representative sequences from OTUs were classified taxonomically using the RDP classifier algorithm (version 2.2) against the Silva (Release 138) 16S rRNA database and the Unite (Release 8.0) ITS rRNA database. For further standardization analysis, each sample was normalized to 28,821 reads (bacterium) and 39,156 reads (fungus) by random selection with the least sequences.

### Statistical analysis

Non-metric multidimensional scaling (NMDS) was performed for the determination of the similarity of bacterial and fungal community based on a Bray–Curtis distance matrix using the “vegan” package in R, and permutational multivariate analysis of variance ordinations (PERMANOVA, 999 permutations) was performed to calculate significant differences in microbial community composition. The interactive effects of type of wetland and season on α-diversity of soil fungal and bacterial communities were analyzed with a two-way repeated measures ANOVA using SPSS version 16.0. Linear discriminant analysis effect size (LEfSe) was performed to detect potential biomarkers from kingdom to species for bacterial and fungal communities based on an LDA threshold score of 4.0. The significance of the abundances at the phylum and class levels was statistically analyzed among soil samples using STAMP software (Parks et al., 2014). The ecological network of bacterial, fungal, and bacterial–fungal communities was constructed based on Pearson’s correlations using the Molecular Ecological Network Analyses (MENA) Pipeline^1^ at the whole level and vegetation type level, respectively (Deng et al., 2012). Topological features were detected, including the total number of nodes, the total number of links, average degree (avgK), average geodesic distance (GD), average cluster coefficient (avgCC), total number of modules, and modularity (Wan et al., 2020). The networks were visualized using Cytoscape version 3.7.1 (Shannon et al., 2003). In networks, the nodes represent OTUs, and the lines represent positive or negative interactions among nodes. The identical color of the nodes in the network is implied from the same phylum. The topological roles of nodes were divided into four categories in these networks based on the within-module connectivity (*Zi*) and among-module connectivity (*Pi*), including network hubs (Zi > 2.5, Pi>0.62), module hubs (Zi > 2.5, Pi≤0.62), connectors (Zi ≤ 2.5, Pi >0.62) and peripherals (Zi ≤ 2.5, Pi≤0.62; Wan et al., 2020). Bacterial and fungal function predictions were annotated using PICRUSt2 and FUNGuild databases (Nguyen et al., 2016; Douglas et al., 2019). The critical predicted KEGG Orthology groups (KOs) of soil bacterial communities was visualized in heatmaps using “Pheatmap” package in R (version 3.6.1). Correlations between bacterial/fungal modules in whole microbial networks and soil nutrient factors were examined using Pearson’s correlation analysis (Deng et al., 2012).

## Results

### Composition and diversity of soil microbial communities

**Figure 1:**
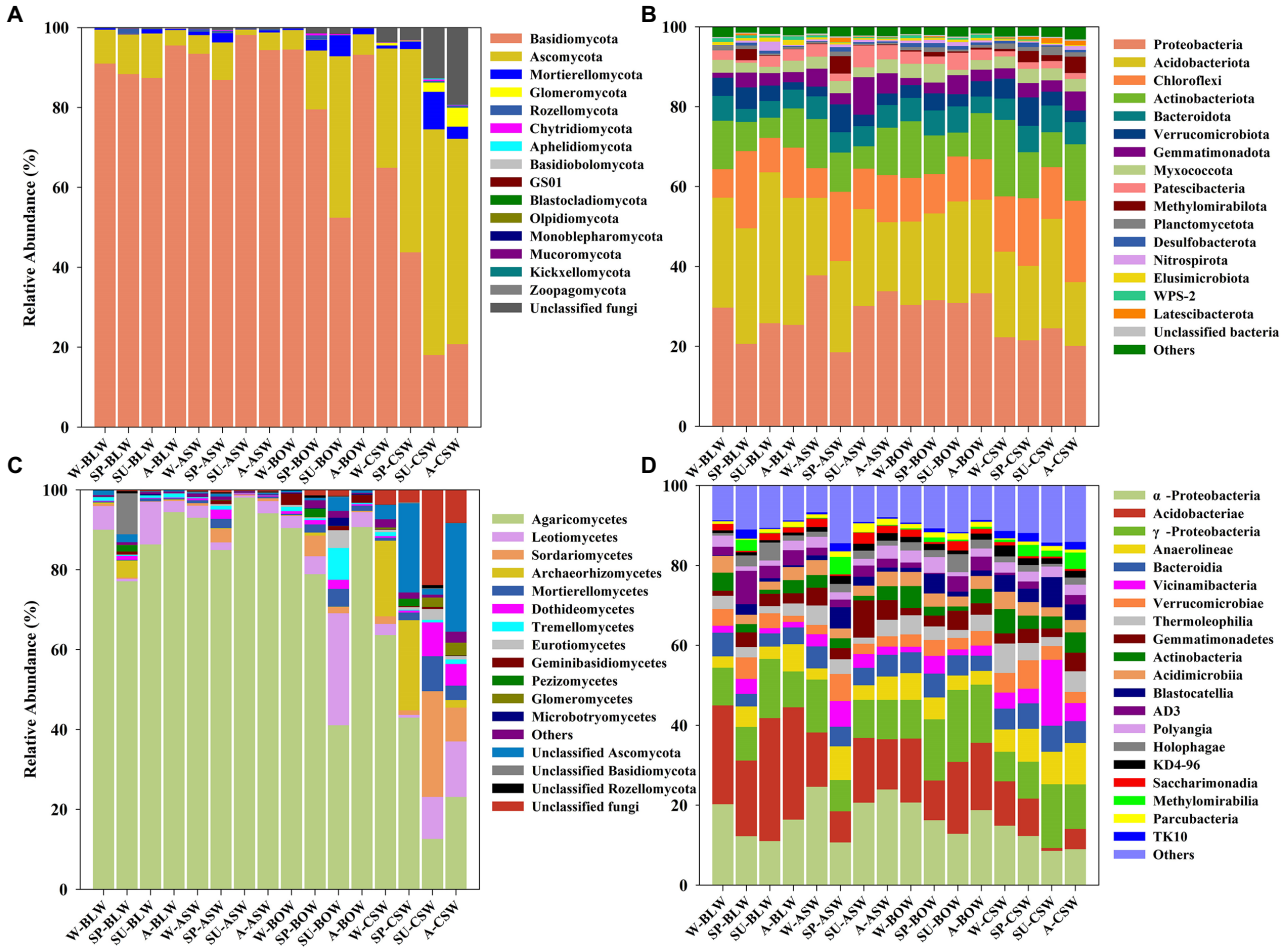
Soil fungal **(A,C)** and bacterial **(B,D)** community composition and relative abundance differences at phylum **(A,B)** and class **(C,D)** levels are shown in circos figures. W, winter; SP, spring; SU, summer; A, autumn.

The soil microbial community compositions from forested wetlands at the phylum and class levels are shown in Figure 1. The identified sequences from the different forested wetland soils were affiliated with 15 fungal phyla. Basidiomycota, Ascomycota, and Mortierellomycota, which accounted for >75.2% of the total soil fungal abundance, were the predominant fungal phyla in wetland soils (Figure 1A). A significantly higher and lower abundance of Basidiomycota and Ascomycota, respectively, were found in *B*. *platyphylla*–*L*. *gmelinii*, *A*. *sibirica*, and *B*. *ovalifolia* wetlands, whereas the complete opposite—a lower and higher abundance of Basidiomycota and Ascomycota, respectively, were mainly found in *C*. *schmidtii* wetland (Supplementary Figure S1). At the class level, 12 dominant classes were identified, as shown in Figure 1C. Agaricomycetes and Leotiomycetes presented a much higher abundance in *B*. *platyphylla*–*L*. *gmelinii*, *A*. *sibirica*, and *B*. *ovalifolia* wetlands, whereas Sordariomycetes, Archaeorhizomycetes, and Mortierellomycetes showed a significantly higher abundance in *C*. *schmidtii* wetland with a significantly lower abundance of *Agaricomycetes* (Supplementary Figure S2).

Forty bacterial phyla were identified from the four wetland soils, seven of which were the dominant phyla, accounting for >83.4% of the total soil bacteria, including Proteobacteria, Acidobacteriota, Chloroflexi, Actinobacteriota, Bacteroidota, Verrucomicrobiota, and Gemmatimonadota (Figure 1B). The distribution of Actinobacteriota in *C*. *schmidtii* wetland was higher, whereas the relative abundance of Acidobacteriota in *B*. *platyphylla*–*L*. *gmelinii* wetland was higher (Supplementary Figure S3). The bacterial communities had a similar taxonomic distribution at the class level across all vegetation types and were dominated by Alphaproteobacteria, Acidobacteriae, Gammaproteobacteria, Anaerolineae, and Bacteroidia. Acidobacteriae, as a predominant class in *C*. *schmidtii* wetland soil, had a much lower abundance than those in other vegetation types (Figure 1D; Supplementary Figure S4). Overall, it appeared that the variation in soil bacterial communities was lower than that of the soil fungal community in all wetland types.

**Figure 2:**
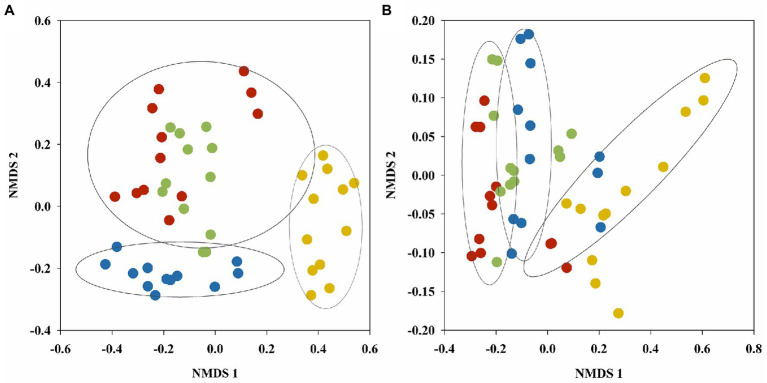
NMDS plots of fungal **(A)** and bacterial **(B)** OTUs found in soil samples of different vegetation types generated using the Bray–Curtis distance matrix. Red, blue, green and yellow dots represent wetlands of *B*. *platyphylla*–*L*. *gmelinii*, *A*. *sibirica*, *B*. *ovalifolia*, and *C*. *schmidtii*, respectively.

The diversity and richness of fungal and bacterial communities was evaluated in the wetland soil *via* Sobs, Shannon, Simpson, and ACE indices (Supplementary Table S1). α- diversity indices of soil fungal and bacterial communities showed significant vegetation type and seasonal effect (except for Shannon and Simpson indices of fungal communities). Using the Bray–Curtis dissimilarity, NMDS analysis was further performed to reveal the fungal and bacterial community variance of the soil samples during the season in the four vegetation types (Figure 2). The NMDS ordination showed that vegetation type primarily affected the fungal (stress: 0.174) and bacterial (stress: 0.096) community distributions of the wetland soils. Soil fungal and bacterial communities both showed clear separation of the three plots according to vegetation type. The samples from *B*. *platyphylla*, *L*. *gmelinii*, and *B*. *ovalifolia* wetlands were clustered together. The distributions of the fungal and bacterial communities from *A*. *sibirica* wetland tended to differ significantly, and the soil fungal and bacterial communities in *C*. *schmidtii* wetland were also far from those of the other samples. In addition, PERMANOVA analysis showed that the vegetation type had a significant effect on the fungal (R^2^ = 0.2239, *p* = 0.001) and bacterial (R^2^ = 0.3000, *p* = 0.001) community structures.

**Figure 3:**
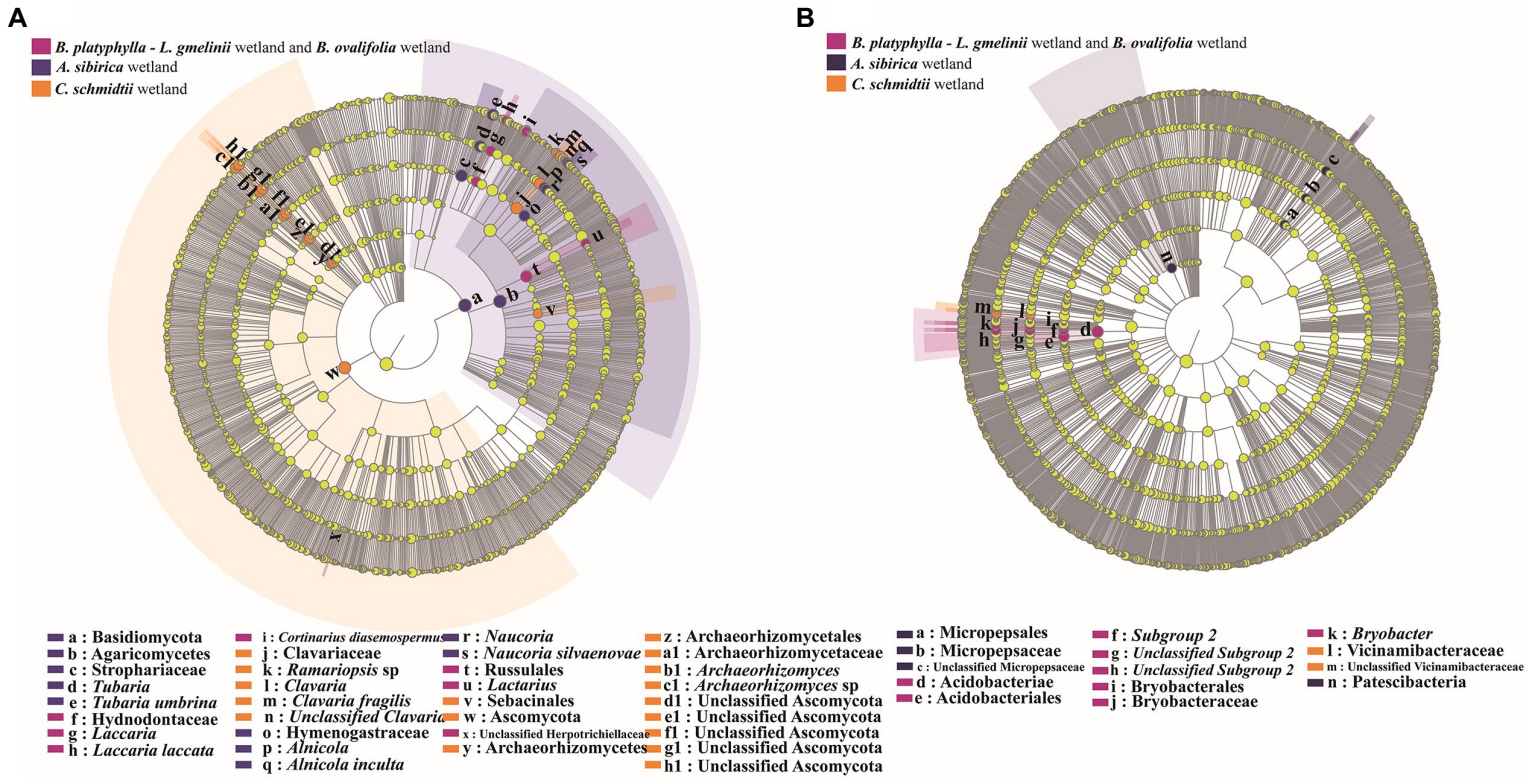
LEfSe analysis of fungal **(A)** and bacterial **(B)** taxa present in the microbiome residing in different types of wetland soils.

According to the results of NMDS, LEfSe analysis was used to distinguish potential discriminating taxa between the three major groups. Considerable differences in taxa are displayed in a cladogram (Figure 3). LEfSe analysis showed 34 indicator taxa (fungi) and 14 indicator taxa (bacteria) among the three groups. Members of fungal taxa in the genera *Laccaria* and *Lactarius* were more enriched in *B*. *platyphylla*–*L*. *gmelinii* and *B*. *ovalifolia* wetlands, whereas those in the genera *Clavaria* and *Archaeorhizomyces* were significantly more abundant in *C*. *schmidtii* wetland. The genera *Tubaria*, *Naucoria*, and *Alnicola* were significantly enriched in *A*. *sibirica* wetland (Figure 3A). Members of bacterial taxa in the genus *Bryobacter* were more enriched in *B*. *platyphylla*–*L*. *gmelinii* and *B*. *ovalifolia* wetlands, whereas those in the family Vicinamibacteraceae were significantly more abundant in *C*. *schmidtii* wetland (Figure 3B). The family Micropepsaceae and phylum Patescibacteria were significantly enriched in *A*. *sibirica* wetland.

### The network characteristics of wetland soil microbial communities

**Figure 4:**
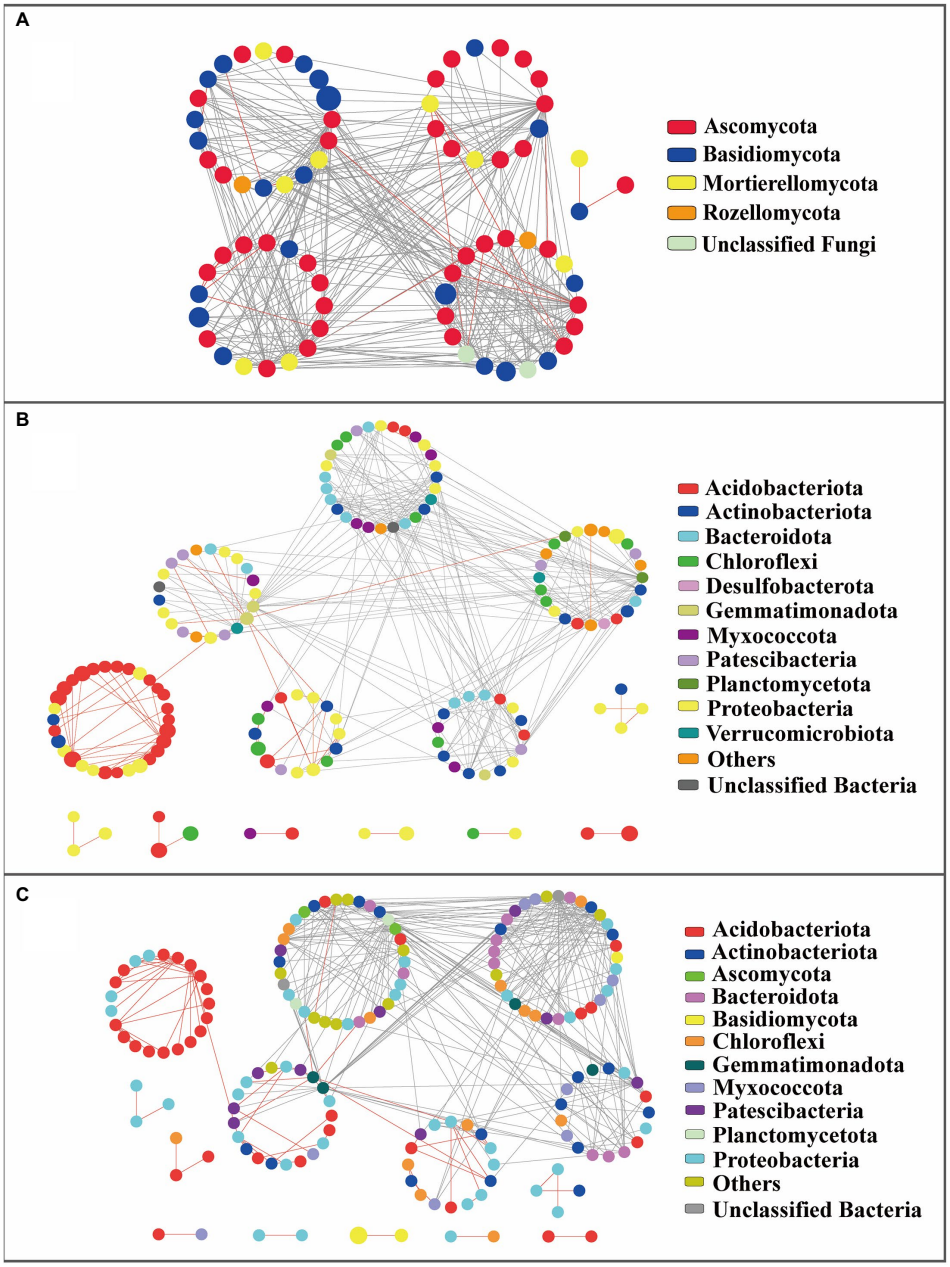
Network analysis of molecular interactions of fungus-fungus **(A)**, bacterium-bacterium **(B)**, or bacterium-fungus **(C)** at whole level. The colors of nodes in the webs indicate the taxa represented by the dots. Red lines represent positive interactions, while gray threads are representatives of negative interplay.

Three association networks were constructed at the whole bacterial, fungal, and bacterial–fungal levels. Respectively 73, 154, and 162 nodes emerged in fungi, bacteria, and bacteria–fungi networks (Table 1). Average path-distance values in empirical networks were higher than the corresponding values in randomized networks, which implies that the networks with closer connections presented small-world behavior. Modularity values from empirical networks were higher than those from corresponding randomized networks, indicating the modular characteristics of the networks. The positive links of the bacteria–fungi community and bacterial community were 79 and 80, respectively, whereas the positive links of the fungal community were only 23 (Figure 4). A total of 319 bacteria–bacteria, 20 bacteria–fungi, and 1 fungi-fungi links were conducive to revealing the interrelationships between two kingdoms from forested wetland soil in the bacteria–fungi network. Twenty bacterial–fungal links were mainly classified as Acidobacteriota–Ascomycota, Patescibacteria–Ascomycota, Proteobacteria–Ascomycota, Chloroflexi–Ascomycota, Bacteroidota–Ascomycota, and Myxococcota–Basidiomycota.

**Table 1:** Whole network properties of microbiome inhabiting forested wetlands soil.

	Network features	Fungi	Bacteria	Fungi- Bacteria
**Empirical networks**	Total nodes	73	154	162
	Total links	332	319	340
	R2 of power-law	0.557	0.886	0.872
	Average degree (avgK)	9.096	4.143	4.198
	Average path distance (GD)	2.309	4.691	4.675
	Average clustering coefficient (avgCC)	0.078	0.040	0.038
	Modularity	0.214	0.495	0.482
**Random networks**	Average path distance (GD)	2.234 ± 0.034	3.32 ± 0.061	3.313 ± 0.063
	Average clustering coefficient (avgCC)	0.322 ± 0.02	0.071 ± 0.012	0.072 ± 0.014
	Modularity	0.206 ± 0.009	0.439 ± 0.008	0.438 ± 0.008

A total of 5, 13, and 14 modules were detected in the corresponding ecological networks of fungi, bacteria, and bacteria–fungi, respectively (Figure 4). In the fungal network, the member of an identified network hub was from Ascomycota. A total of 32 fungal connectors belonged to Ascomycota, Basidiomycota, Mortierellomycota, Rozellomycota, and unclassified fungi (Supplementary Table S2). Members of Actinobacteriota (33.3%) and Bacteroidota (33.3%) dominated connectors from the bacterial networks. Four network hubs in the bacterial network were identified, and only one module hub was detected. A total of 16 connectors in the bacteria–fungi network belonged to Actinobacteriota, Bacteroidota, Patescibacteria, Proteobacteria, Myxococcota, Gemmatimonadota, and Chloroflexi, four network hubs were found.

Microbial networks at vegetation type-level also showed modularity features (Supplementary Table S3). All of these networks possessed small-world features similar to the fungal and bacterial networks at the whole level. The fungal and bacterial networks at the whole level had fewer positive links than the corresponding networks at vegetation type-level. Bacterial networks tended to have more positive correlations than those of fungal networks. The microbial networks from forested and shrub wetlands had more positive links than those from herbaceous vegetation wetlands (Supplementary Figures S5, S6). The links in vegetation type-level network relationships connected more tightly with each other. Network hubs were only identified in the fungal network of *A*. *sibirica* wetland (OTU2151 and OTU356) and the bacterial network from *C*. *schmidtii* wetland (OTU3; Supplementary Tables S4, S5).

### Functional profile of soil fungal and bacterial communities

**Figure 5:**
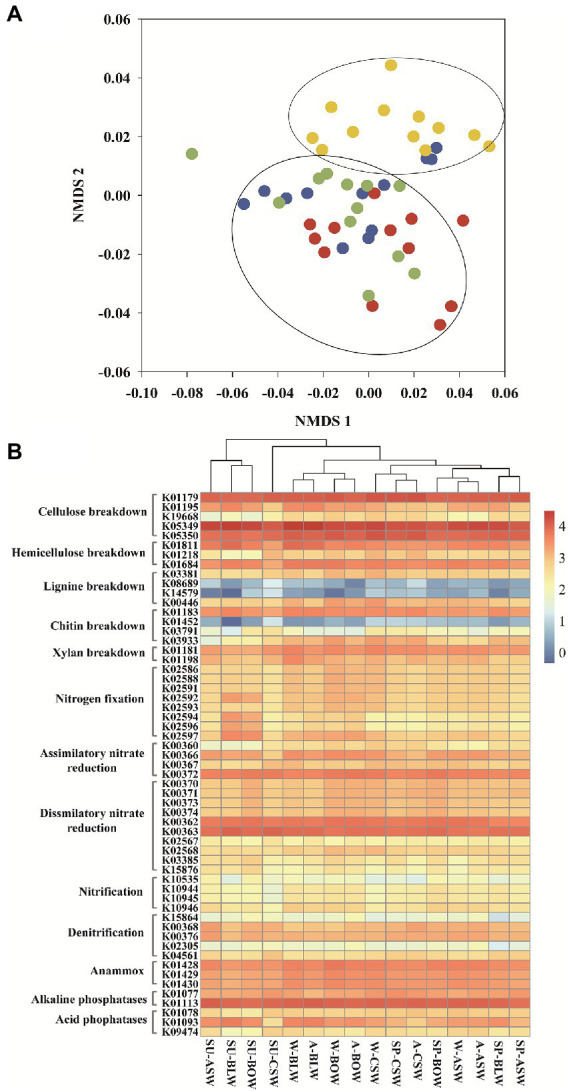
**(A)** NMDS plots of predicted bacterial enzymes found in microbiome inhabiting soils of different vegetation types generated by using the Bray-Curtis distance matrix. Red, blue, green and yellow dots represent wetlands of *B*. *platyphylla*-*L*. *gmelinii*, *A*. *sibirica, B*. *ovalifolia* and *C*. *schmidtii*, respectively. **(B)** Comparison of assumed KEGG Orthology groups (KOs) of soil bacterial communities existing in distinctive forested wetlands.

Various functional characteristics were predicted from bacterial OTUs using the PICRUSt2. A total of 7, 227 functional KEGG orthologs (KOs) and 426 enzymes were acquired from the predicted functional profiles. These enzymes and KOs can predict the potential capacity of energy metabolism and substance degradation from wetland soil bacteria. The overall distributions of the bacterial enzymes predicted based on EC were visualized in the NMDS plots (stress: 0.120; Figure 5A). Wetland vegetation types resulted in different distributions of the bacterial functional enzymes. The type and abundance of enzymes were similar among *B*. *platyphylla*–*L*. *gmelinii*, *B*. *ovalifolia*, and *A*. *sibirica* wetlands and were clustered together, whereas the distributions of bacterial enzymes from *C*. *schmidtii* wetland were far from those of the other samples.

We further described the critical KOs involved in nutrient cycling. A total of 57 key KOs participated in 13 metabolic pathways, including those of cellulose, hemicellulose, lignin, chitin, and xylan breakdown, nitrogen fixation, assimilatory nitrate reduction, dissimilatory nitrate reduction, nitrification, denitrification, anammox, alkaline phosphatases, and acid phosphatases. Cluster analysis of different wetlands was carried out according to these key functional KOs (Figure 5B). The results showed that soil samples from *B. platyphylla*–*L. gmelinii*, *B. ovalifolia*, and *A. sibirica* wetlands in summer were gathered together. Furthermore, the soil samples in *C*. *schmidtii* wetland group with low nitrification and nitrogen fixation were clustered together, whereas other vegetation types exhibited higher functional potentials of nitrification, nitrogen fixation, and acid phosphatases (K01093) than those in *C*. *schmidtii* wetland.

**Figure 6:**
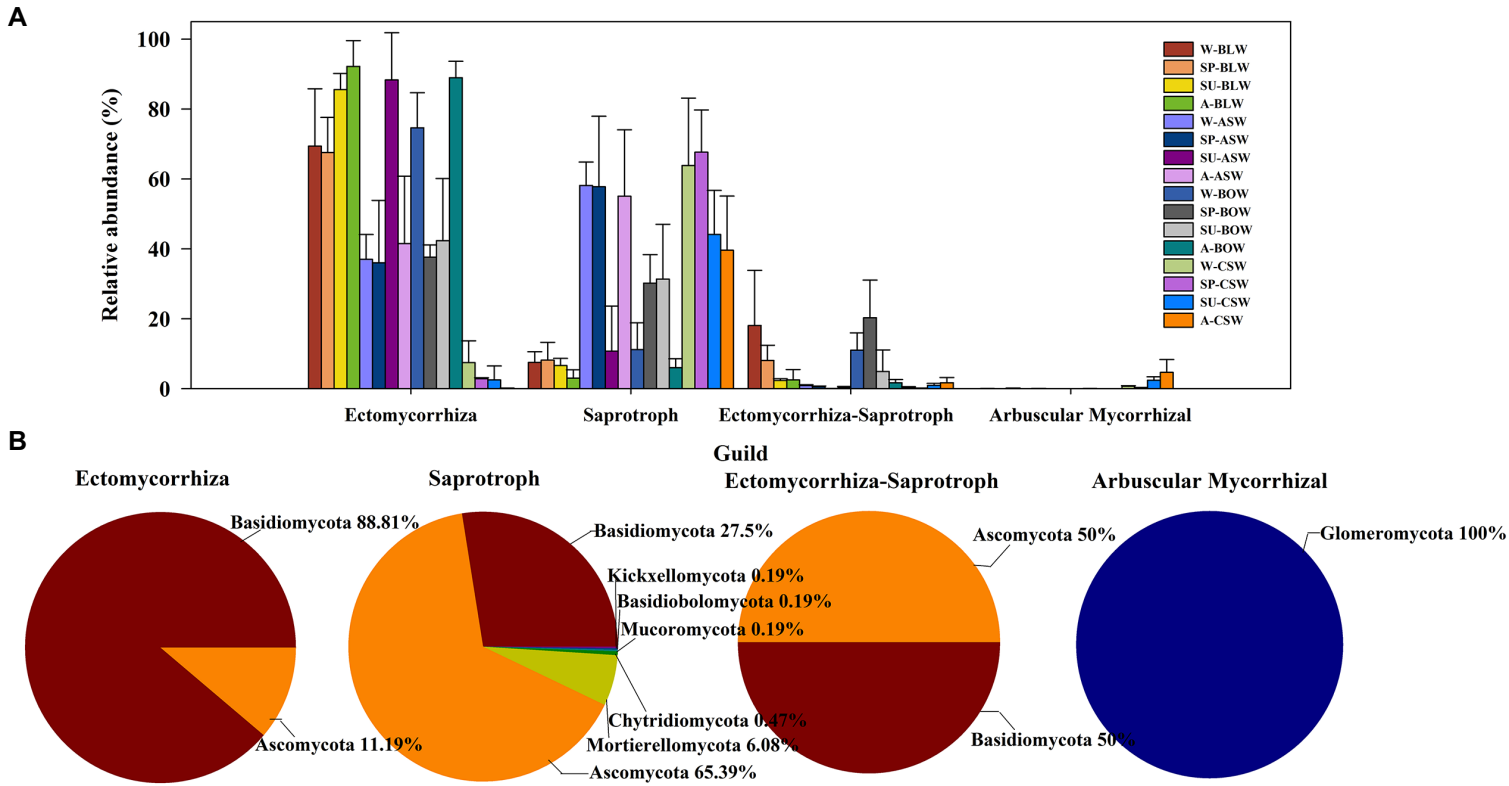
**(A)** The relative abundance of soil fungi belonging to the four functional groups. **(B)** The compositions of the four functional categories of soil fungi at the phylum level.

In this study, most fungi were mainly divided into ectomycorrhizal (EcM), saprotrophic, EcM-Saprotroph, and arbuscular mycorrhizae (AM) taxa. A total of 277 EcM fungal OTUs, 1,069 saprotrophic fungal OTUs, 96 EcM-Saprotroph fungal OTUs, and 127 AM fungal OTUs were detected. The EcM, saprotrophic, EcM-Saprotroph, and AM fungal communities were compared among the different wetland soils according to their total abundance (Figure 6A; Supplementary Figure S7). The total abundance of EcM fungi was significantly higher in soil samples of *B*. *platyphylla*–*L*. *gmelinii*, *A*. *sibirica*, and *B*. *ovalifolia* wetlands than in those of *C*. *schmidtii* wetland. The total abundance of EcM-Saprotroph fungi was significantly higher in the soil of *B*. *platyphylla*–*L*. *gmelinii* and *B*. *ovalifolia* wetlands than in those of other vegetation types. Additionally, saprotrophic fungi showed significantly higher total abundance in the soils of *C*. *schmidtii* and *A*. *sibirica* wetlands than in those of other vegetation types, whereas AM fungal communities were mainly found in *C*. *schmidtii* wetland. Regarding functional fungal OTU-based analysis, most EcM fungal OTUs belonged to the phyla Basidiomycota (88.81%) and Ascomycota (11.19%; Figure 6B). Saprotrophic fungal OTUs were mainly classified as Ascomycota (65.39%), followed by Basidiomycota (27.50%), Mortierellomycota (6.08%), and Chytridiomycota (0.47%). All AM fungal OTUs were from the phylum Glomeromycota. The proportion of the phylum Basidiomycota and Ascomycota in EcM-Saprotroph fungal OTUs was approximately 1:1.

### Correlations of network modules with soil nutrient properties

The relevance of soil nutrient properties (Supplementary Figure S8) to microbial network modules (the small modules were ignored with less than 5 members) was also assessed. In fungal communities, network modules were significantly positively correlated with TN and water-soluble K (Table 2). No significant correlation with any of the nutrient properties was detected in the fungal module I. In bacterial networks, most of the modules positively correlated with TN, water-soluble K, water-soluble Mg, and water-soluble Na. However, bacterial module I and II were significantly negatively correlated with soil nutrient properties. Water-soluble Zn were also not significantly correlated with most bacterial modules. In general, the bacterial network modules were more closely related to soil nutrients than fungal networks.

**Table 2:** Correlations between soil nutrient properties and soil microbial community network modules.

	Module	TN		K		Na		Ca		Mg		Zn	
		*r*	*p*	*r*	*p*	*r*	*p*	*r*	*p*	*r*	*p*	*r*	*p*
Fungal community	I	0.23	0.1	−0.027	0.9	0.036	0.8	0.15	0.3	0.071	0.6	−0.16	0.3
	II	0.45	0.001	−0.053	0.7	−0.11	0.4	−0.25	0.09	−0.055	0.7	−0.1	0.5
	III	0.62	<0.001	0.36	0.01	0.18	0.2	−0.097	0.5	0.065	0.7	−0.33	0.02
	IV	0.54	<0.001	0.25	0.08	0.29	0.04	−0.19	0.2	0.046	0.8	−0.15	0.3
Bacterial community	I	−0.81	<0.001	−0.36	0.01	−0.42	0.003	−0.054	0.7	−0.38	0.007	0.24	0.1
	II	−0.68	<0.001	−0.44	0.002	−0.39	0.006	−0.05	0.7	−0.36	0.01	0.31	0.03
	III	0.58	<0.001	0.49	<0.001	0.34	0.02	0.31	0.03	0.61	<0.001	−0.2	0.2
	IV	0.7	<0.001	0.45	0.001	0.35	0.01	0.12	0.4	0.47	<0.001	−0.18	0.2
	V	0.72	<0.001	0.4	0.005	0.37	0.01	−0.049	0.7	0.3	0.04	−0.23	0.1
	VI	0.54	<0.001	0.5	<0.001	0.43	0.002	0.12	0.4	0.46	0.001	−0.29	0.04

## Discussion

The plant species and seasonal variations can change the composition and structures of soil microbial community (Koranda et al., 2013; Li et al., 2020; Yang et al., 2020). Our results indicated that vegetation type and season have an effect on shifting soil community compositions at forested wetland ecotone, but vegetation type was the main factor driving the alterations of the diverseness and functionalities of bacterial and fungal communities. This may be attributed to the fact that soil microbial communities are likely to be regulated with the root exudates and leaf litter of the plant species (Reinhold-Hurek et al., 2015; Schappe et al., 2017; Fu et al., 2020). He et al. (2020) also reported that vegetation type could be a major factor affecting soil properties by regulating plant-associated microbial communities, compared to the season. The patterns of these responding microbes can be conducive to the understanding of the characteristics of vegetation; hence, we surmised that the dominant microbes related to the different vegetation types could be used as indicators.

The existence of a microbial community with specialized functions is essential for maintaining the stability of soil ecosystem (Widdig et al., 2020). Arbuscular mycorrhizal fungi (AMF) and ectomycorrhizal fungi (EcM), which are two important taxa of mycorrhizal fungi, can form symbiotic relationships with roots of many plants. This association helps host plants absorb soil nutrients and water (van der Heijden et al., 2015). In this study, soil samples from *C*. *schmidtii* wetland with a higher abundance of AMF had a lower distribution of EcM, whereas soil samples from *B*. *platyphylla*–*L*. *gmelinii*, *A*. *sibirica*, and *B*. *ovalifolia* wetlands showed the complete opposite result. This finding revealed that vegetation types presented a trend from EcM vegetation to AMF vegetation along with forested wetland ecotones. In soils, the dominant change in either AMF or EcM could imply a variation in microbially mediated processes, which may further influence plant growth and plant–soil feedback (Nash et al., 2020). EcM and AMF, as nutrient conduits, can efficiently absorb and transport nutrients to their host plants but cannot degrade litter (Cheeke et al., 2017; Fang et al., 2020). However, saprotrophic fungi are the major decomposers of carbon during litter decomposition (e.g., lignin), relying on their specific carbohydrate-hydrolytic enzymes (Hobbie and Horton, 2007; Asplund et al., 2018; Godin et al., 2019). It was found that saprotrophic fungi might be restrained by EcM as a result of competition for nitrogen resources, which may explain the differences in the abundance of soil saprotrophic fungi between EcM and AMF vegetation (Fernandez and Kennedy, 2016). Different fungal taxa with overlapping niches can compete for soil resources; however, these interactions also further adjust the assemblages of the microbial community (Bodeker et al., 2016; Mujic et al., 2016; Perez-Valera et al., 2018). Although soil functional fungi were the critical drivers of soil nutrient cycling in this study, the role of soil bacteria should not be ignored.

Soil bacterial communities were further analyzed based on the 16S rRNA gene-predicted functional analysis. In this study, the clustering of the abundance of N and P cycling-related microbial KOs and litter breakdown KOs mainly appears the distribution characteristics of vegetation. N is an essential element in terrestrial ecosystems, and its cycling is driven mainly by N cycling-associated microbial community (Damashek and Francis, 2018; Du et al., 2020). In this study, the low relative abundance of nitrogen fixation-associated KOs in *C. schmidtii* wetland was detected, especially for KO2594 and KO2596. In addition, we also observed that potential nitrification-associated KOs was low in *C*. *schmidtii* wetland, but the abundance of nitrogen reduction- and denitrification-associated KOs showed an increasing trend. These findings were in accordance with the relative variation in soil total N, along with changes in vegetation. Similarly, phosphatases secreted by soil microorganisms are very important in P cycling, forming inorganic P that is absorbed by plants and microorganisms (Qian et al., 2007). Our study revealed that phosphatase-associated KOs, especially KO1093, were found in lower abundances in *C*. *schmidtii* wetland than in other wetlands. Litter decomposition is a key process that provides nutrients for the growth of plants and soil microorganisms (Cusack et al., 2009; Garcia-Palacios et al., 2016). We also found that cellulose and hemicellulose breakdown in litter breakdown-associated KOs was higher, whereas lignine and chitin breakdown was lower. The overall distributions of the bacterial enzymes predicted based on EC numbers also showed that soil samples from *C. schmidtii* wetland were gathered apart from those of the other wetlands.

Microbial network associations and co-occurrence structures in complex microbial communities are often related to the adaptation to environmental stress (Li et al., 2017; Asemaninejad et al., 2020). Topological features and keystone species of soil fungal and bacterial association networks in forested wetlands were further explored. In this study, microbial ecological networks clearly showed that some OTUs (e.g., hubs or connectors) had many links with other OTUs or species. These hubs, as keystone taxa, are important for maintaining the structure and integrity of the microbial community, which would create a drastic change in community composition and function if they were removed (van der Heijden and Hartmann, 2016; Banerjee et al., 2018). Furthermore, there were direct and indirect linkages between some fungi and bacteria in the fungi–bacteria network, indicating that the fungal communities may be likely to influence bacterial communities. It was found that certain bacteria could rely on secondary metabolites secreted by fungi to survive (Duponnois and Kisa, 2006). The specific bacterial communities may also utilize fungal hyphae (e.g., EcM) as a transport vector to move and disperse in the surrounding soil, although these bacteria may not thrive alone in such environment (Vik et al., 2013). Overall, the keystone species may trigger niche competition to change the composition of the microbial community by secreting certain metabolites, and also adjust the abundance of their partners through a synergistic effect (van der Heijden et al., 1998; Kommineni et al., 2015).

At vegetation type-level, the fungal and bacterial modularity in *C. schmidtii* wetland was lower than that of other vegetation types. The modularity of the microbial community can contribute to reducing the responses of soil microbes to environmental disturbance (Kitano, 2004; Wan et al., 2020). Thus, it is possible that soil microbial communities in *B. platyphylla*–*L. gmelinii*, *A. sibirica*, and *B. ovalifolia* wetlands could remain more stable when confronted with environmental disturbances. The compartmentalization with more modules at vegetation type level was relatively high compared with those at the overall level (Olesen et al., 2007). Meanwhile, topological properties of fungal and bacterial networks also emerged as characteristics of small world, which are propitious to microbial communities to rapidly deal with the variation of the environment (Montoya et al., 2006; Fang et al., 2020). Ten network hubs/module hubs, identified as EcM or saprotrophic fungi, were detected at vegetation type-level fungal networks, which vary along different wetland vegetation types. This indicates that keystone species may not always exist in a variable environment (Banerjee et al., 2018). The variety of vegetation influences the structure and composition of microbial communities in the soil environment, and thus certain keystone species might only exist in a specific vegetation soil. Similarly, 52 network hubs/module hubs were detected in vegetation type-level bacterial network, exceeding the count of fungal hubs. However, these hubs (OTUs) did not recur among different wetland soils, except for OTU3554 and OTU318, which was similar to fungal vegetation type-level networks. However, these hubs, as keystone species, had lower relative abundance. This result was in line with previous findings on keystone species without numerical dominance (Chao et al., 2016; Comte et al., 2016; Hill et al., 2016). Positive interactions in the microbiome enlarge the environmental range of their existence and broaden their niche occupancy through positive feedback and cooscillation (Stachowicz, 2001; Coyte et al., 2015). More positive interactions were detected in three wetland soils in vegetation type-level fungal and bacterial networks compared with those in *C*. *schmidtii* wetland soil, which implied that it was more beneficial for the three wetland vegetation types to adapt to the changing environment according to the powerful microbial network.

Vegetation type and season may also have an indirect impact on soil microbial community composition by changing soil nutrients (Han et al., 2021). In this study, we detected that multiform bacterial and fungal communities from different modules of ecological networks varied distinctly in terms of their correlations with soil nutrients. Most fungal and bacterial modules are compelled by similar driving factors. This result indicates that the microbial communities from different modules were inclined to prefer similar habitats. Among these soil nutrient properties, TN and water-soluble K were identified as the most critical drivers. It has previously been shown that soil N and K content could be key factors in the diversity and structure of soil microbial communities (Li et al., 2015; Liang et al., 2021). In this study, most of the soil bacterial modules showed a positive correlation with mineral elements in soil nutrient factors. Uroz et al. (2012) also found that mineral elements, as a key driving force, could influence the bacterial community. Soil bacterial communities were more closely positively or negatively correlated with soil nutrients, compared with soil fungal communities. Similar soil nutrients but different degrees of correlation with microbial communities might imply that bacterial and fungal communities occupy different niches, which is beneficial for the decrease in competition under identical habitats (Wei et al., 2020). By exploring the relationships of different ecological network modules of bacterial and fungal communities with soil nutrient factors, our results indicate that microbial network stability and keystone species play an important role in soil nutrient cycling, and soil nutrients are also essential for shaping the diversity and composition of soil microbial communities.

## Conclusion

Through a deep analysis of the distribution patterns of soil bacterial and fungal communities from forested wetland ecotones, we revealed vegetation as the primary driving factor for variation in soil bacterial and fungal community composition, diversity, and function. Our results demonstrate that both bacteria and fungi play critical roles in forested wetland soil ecosystems, and their interaction with plants is essential for regulating plant growth, driving soil element cycles, and maintaining ecological balance. In our study, the vegetation species from forested and shrub wetlands established mostly ectomycorrhizal associations, and the species from herbaceous vegetation wetlands such as *C*. *schmidtii* wetland established arbuscular associations. Bacterial functional predictions also demonstrated that potential microbial functions from *C*. *schmidtii* wetland soil have more obvious differences than other wetland vegetation in forested wetland ecotones. The co-occurrence networks of soil bacteria and fungi were more driven by mutualism (positive correlations) in forested and shrub wetland ecosystems than in *C*. *schmidtii* wetland. Overall, the change in biological co-occurrence patterns caused by the transformation of plant–fungal–bacterial interactions may drive the plant diversity pattern of forested wetland ecotones, which provides a new perspective to have great implications for preserving the stability and restoration of the forested wetland ecotones.

## Data availability statement

All bacterial and fungal raw sequences have been deposited in the NCBI Sequence Read Archive, accession number SRP304662.

## Author contributions

DW, HB, and BN designed the experiments. DW, CZ, and QC performed the experiments. DW, MP, MH, YD, FG, and QZ analyzed the data. DW and HB wrote the manuscript. BN and MH revised the manuscript. All authors approved the final version of manuscript.

## Funding

This work was supported by the National Natural Science Foundation of China (No. 31500508), Natural Science Foundation of Heilongjiang Province (No. LH2020C041), the Fundamental Research Funds for the Central Universities (No. 2572020BD02), and Science and Technology Development Project of National Forestry and Grassland Administration (No. KJZXSA202034).

## Conflict of interest

The authors declare that the research was conducted in the absence of any commercial or financial relationships that could be construed as a potential conflict of interest.

## Publisher’s note

All claims expressed in this article are solely those of the authors and do not necessarily represent those of their affiliated organizations, or those of the publisher, the editors and the reviewers. Any product that may be evaluated in this article, or claim that may be made by its manufacturer, is not guaranteed or endorsed by the publisher.

## Supplementary material

The Supplementary material for this article can be found online at: https://www.frontiersin.org/articles/10.3389/fmicb.2023.1160683/full#supplementary-material

## Footnotes

﻿^1^https://ieg4.rccc.ou.edu/mena
