# Supplementary material for: The characteristics of soil microbial co-occurrence networks across a high-latitude forested wetland ecotone in China
Source: Front Microbiol. 2023 Mar 21;14:1160683. doi: 10.3389/fmicb.2023.1160683 (PMC10072330; doi:10.3389/fmicb.2023.1160683)
Supplement: Supplementary file 1 [file Data_Sheet_1.docx]

**Supplementary Material**

**The characteristics of soil microbial co-occurrence networks across a high-latitude forested wetland ecotone in China**

Di Wu^1,2,3a^, Hui Bai^4a^, Caihong Zhao^3^, Mu Peng^3^, Qi Chi^3^, Yaping Dai^1,3^, Fei Gao^1,3^, Qiang Zhang^5^, Minmin Huang^5^*, Ben Niu^1,2,3^*

^1^State Key Laboratory of Tree Genetics and Breeding, Northeast Forestry University, Harbin 150040, China

^2^The Center for Basic Forestry Research, College of Forestry, Northeast Forestry University, Harbin 150040, China

^3^College of Life Science, Northeast Forestry University, Harbin 150040, China

^4^Key Laboratory of Fast-Growing Tree Cultivating of Heilongjiang Province, Forestry Science Research Institute of Heilongjiang Province, Harbin 150040, China

^5^Institute of Economic Forest of Xinjiang Academy of Forestry Sciences, Urumqi 830063, China

***Corresponding author**: Ben Niu: ben_niu@nefu.edu.cn

Minmin Huang: 30481658@qq.com

**^a^ These authors contributed equally to this work.**

**Table S1** α-diversity indices of soil fungal and bacterial community from four vegetation types in forest-wetland ecotone of northern Xiaoxing'an Mountains spanning different seasons.

| Sample | Fungal community | | | | | | | | Bacterial community | | | | | | | |
| --- | --- | --- | --- | --- | --- | --- | --- | --- | --- | --- | --- | --- | --- | --- | --- | --- |
|  | Sobs | | Shannon | | Simpson | | ACE | | Sobs | | Shannon | | Simpson | | ACE | |
| W-BLW | 267±12 | | 2.12±0.13 | | 0.27±0.03 | | 470.69±93.37 | | 1742±46 | | 6.17±0.02 | | 0.005±0.0002 | | 2187.70±54.84 | |
| W-ASW | 357±14 | | 2.06±0.22 | | 0.30±0.06 | | 514.92±19.85 | | 2086±263 | | 6.35±0.21 | | 0.004±0.0009 | | 2746.69±351.46 | |
| W-BOW | 286±59 | | 2.74±0.53 | | 0.13±0.08 | | 472.68±19.46 | | 2132±163 | | 6.32±0.27 | | 0.006±0.0033 | | 2734.77±183.82 | |
| W-CSW | 489±59 | | 3.19±0.36 | | 0.11±0.04 | | 718.35±69.35 | | 2481±112 | | 6.54±0.03 | | 0.004±0.0002 | | 3211.56±226.22 | |
| SP-BLW | 363±100 | | 3.20±0.08 | | 0.09±0.01 | | 500.49±103.17 | | 1882±121 | | 6.06±0.11 | | 0.006±0.0006 | | 2552.05±166.51 | |
| SP-ASW | 499±177 | | 2.16±0.78 | | 0.33±0.19 | | 627.74±114.08 | | 2461±210 | | 6.53±0.16 | | 0.004±0.0009 | | 3249.96±252.97 | |
| SP-BOW | 655±100 | | 3.38±0.53 | | 0.11±0.04 | | 785.63±58.06 | | 2384±30 | | 6.51±0.04 | | 0.004±0.0002 | | 3113.51±66.70 | |
| SP-CSW | 350±36 | | 2.73±0.47 | | 0.16±0.08 | | 525.96±79.03 | | 2453±129 | | 6.55±0.05 | | 0.004±0.0001 | | 3206.17±132.97 | |
| SU-BLW | 209±41 | | 1.83±0.30 | | 0.31±0.05 | | 259.61±39.73 | | 1704±106 | | 5.85±0.09 | | 0.009±0.0005 | | 2199.56±174.05 | |
| SU-ASW | 124±29 | | 1.21±0.55 | | 0.53±0.25 | | 182.14±14.82 | | 2005±88 | | 6.18±0.08 | | 0.006±0.0012 | | 2570.95±139.38 | |
| SU-BOW | 266±63 | | 3.19±0.89 | | 0.10±0.06 | | 298.00±30.03 | | 1957±104 | | 6.08±0.13 | | 0.007±0.0013 | | 2500.62±109.63 | |
| SU-CSW | 513±20 | | 4.00±0.41 | | 0.06±0.04 | | 572.81±24.75 | | 2219±174 | | 6.61±0.12 | | 0.003±0.0005 | | 2642.73±209.36 | |
| A-BLW | 221±52 | | 2.04±0.43 | | 0.25±0.09 | | 348.79±60.58 | | 1978±42 | | 6.20±0.06 | | 0.005±0.0006 | | 2603.85±117.91 | |
| A-ASW | 238±14 | | 2.18±0.19 | | 0.22±0.09 | | 339.67±55.76 | | 2157±149 | | 6.39±0.12 | | 0.004±0.0006 | | 2775.31±116.69 | |
| A-BOW | 251±40 | | 2.55±0.21 | | 0.15±0.03 | | 363.39±23.26 | | 2024±54 | | 6.22±0.10 | | 0.005±0.0011 | | 2650.08±88.48 | |
| A-CSW | 385±155 | | 3.21±0.95 | | 0.16±0.17 | | 451.91±180.04 | | 2299±274 | | 6.47±0.24 | | 0.004±0.0013 | | 2915.51±276.30 | |
|  | F | P | F | P | F | P | F | P | F | P | F | P | F | P | F | P |
| Season | 19.331 | <0.001 | 1.199 | 0.331 | 1.088 | 0.373 | 41.546 | <0.001 | 8.320 | 0.001 | 5.383 | 0.006 | 5.583 | 0.005 | 16.025 | <0.001 |
| Season×Vegetation type | 8.401 | <0.001 | 3.026 | 0.015 | 2.081 | 0.073 | 7.365 | 0.002 | 1.421 | 0.234 | 2.011 | 0.083 | 2.422 | 0.040 | 1.886 | 0.104 |
| Vegetation type | 7.515 | 0.01 | 33.657 | <0.001 | 18.854 | 0.001 | 10.166 | 0.004 | 53.790 | <0.001 | 51.836 | <0.001 | 20.335 | <0.001 | 37.994 | <0.001 |


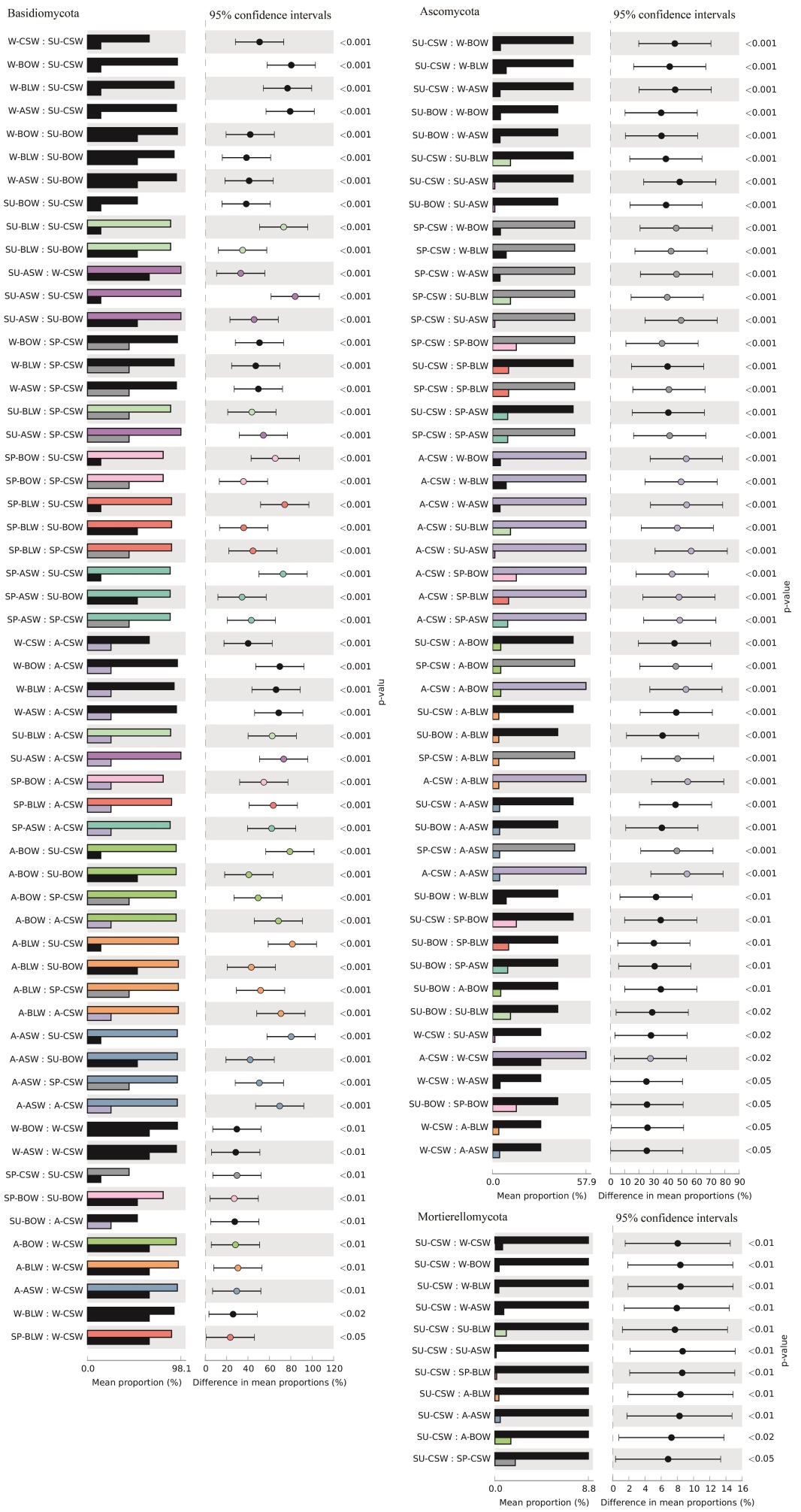


**Fig. S1.** The significance test of the relative abundance of soil fungal community at phylum level from different wetlands.


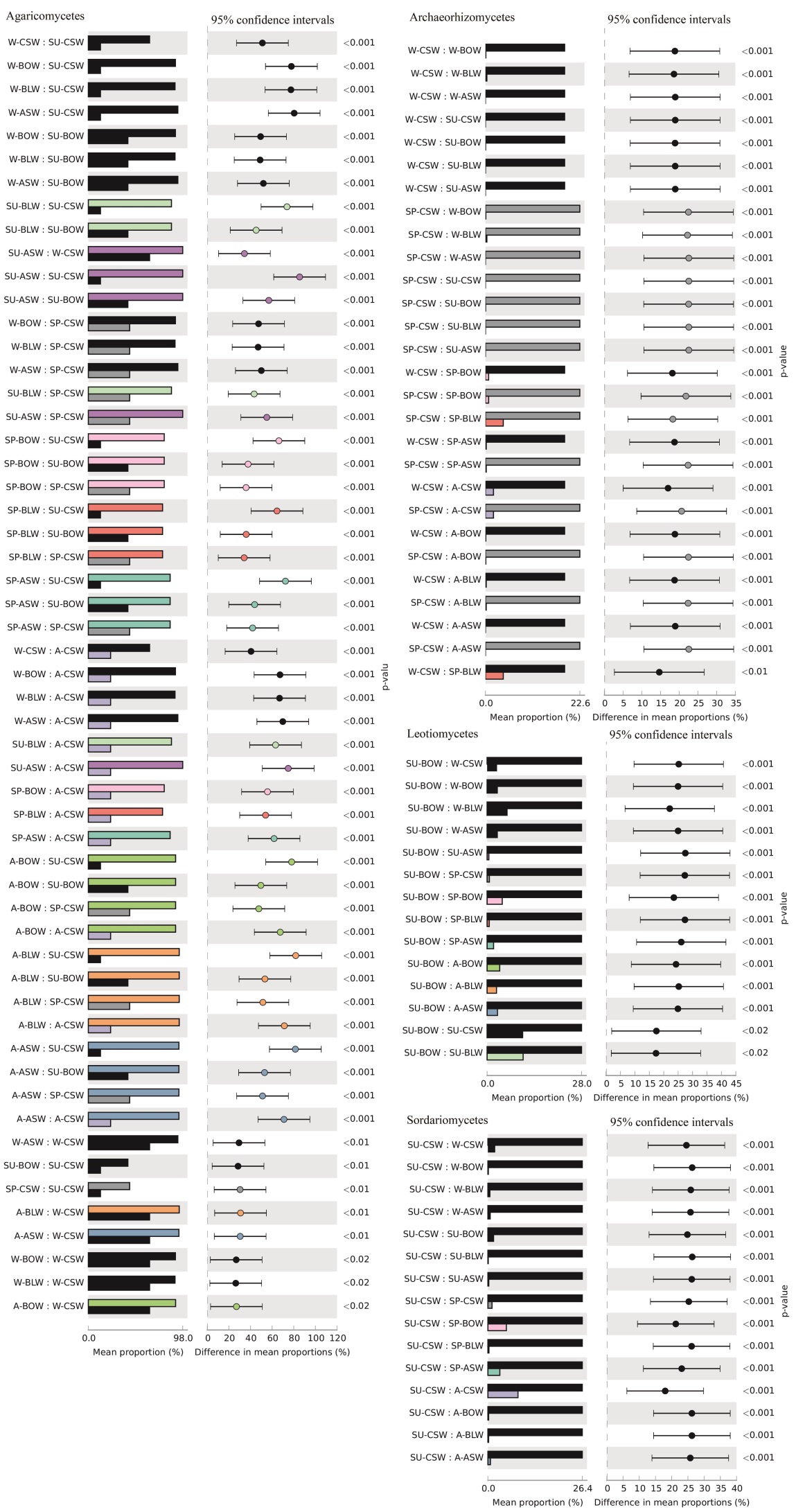


**Fig. S2.** The significance test of the relative abundance of soil fungal community at class level from different wetlands.


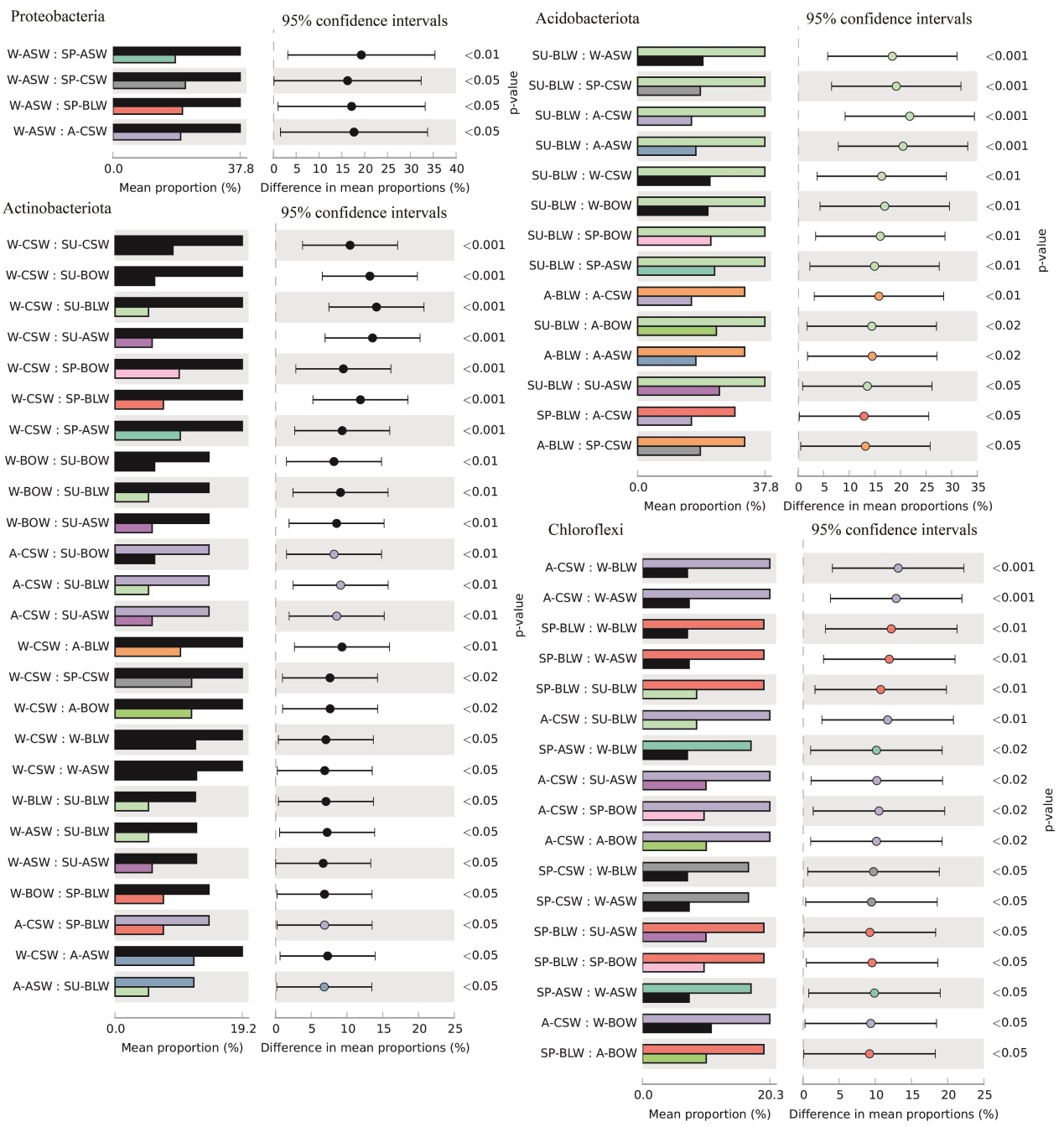


**Fig. S3.** The significance test of the relative abundance of soil bacterial community at phylum level from different wetlands.


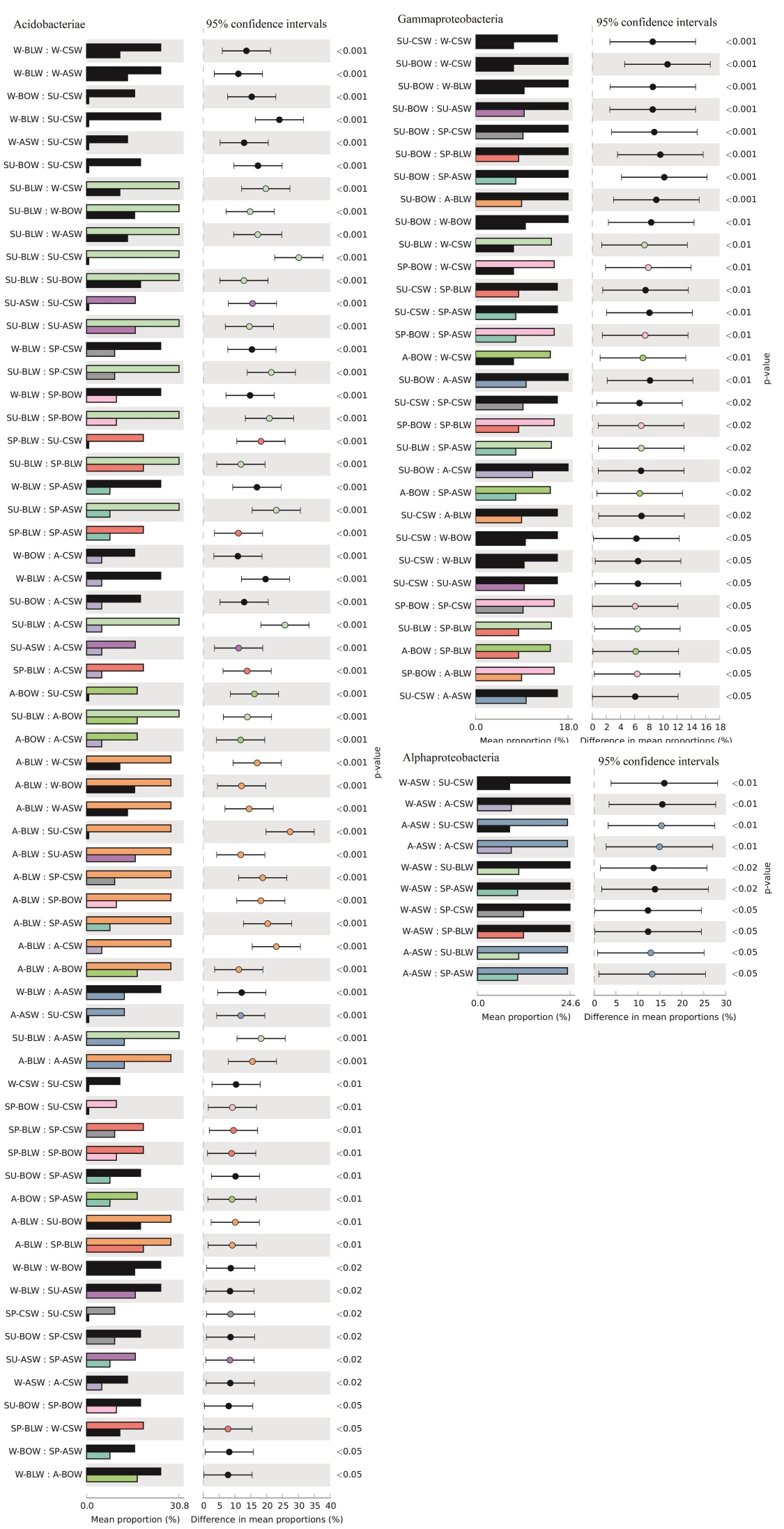


**Fig. S4.** The significance test of the relative abundance of soil bacterial community at class level from different wetlands.

**Table S2** Network hubs, module hubs and connectors in the networks within whole bacterial, fungal and bacterial-fungal communities.

|  |  | OTU | Phylum | Genus | Funguild | OTU | Phylum | Genus | Funguild |
| --- | --- | --- | --- | --- | --- | --- | --- | --- | --- |
| Fungi | Network hub | OTU2837 | Ascomycota | *Pezizella* | Ectomycorrhizal- Saprotroph |  |  |  |  |
|  | connector | OTU337 | Ascomycota | Unclassified Helotiales | - | OTU3044 | Mortierellomycota | *Mortierella* | Saprotroph |
|  |  | OTU420 | Ascomycota | *Trichoderma* | Saprotroph | OTU739 | Ascomycota | *Phialea* | Saprotroph |
|  |  | OTU755 | Ascomycota | *Cenococcum* | Ectomycorrhizal | OTU2087 | Mortierellomycota | *Mortierella* | Saprotroph |
|  |  | OTU275 | Mortierellomycota | *unclassified Mortierellace* | Saprotroph | OTU125 | Unclassified Fungi | Unclassified Fungi | - |
|  |  | OTU3070 | Basidiomycota | *Cortinarius* | Ectomycorrhizal | OTU12 | Basidiomycota | *Laccaria* | Ectomycorrhizal |
|  |  | OTU2688 | Ascomycota | *Exophiala* | Saprotroph | OTU307 | Basidiomycota | *Laccaria* | Ectomycorrhizal |
|  |  | OTU503 | Ascomycota | Unclassified Helotiales | - | OTU617 | Ascomycota | *Ilyonectria* | Saprotroph |
|  |  | OTU25 | Basidiomycota | *Laccaria* | Ectomycorrhizal | OTU2998 | Rozellomycota | Unclassified Rozellomycota | - |
|  |  | OTU74 | Basidiomycota | *Entoloma* | Ectomycorrhizal- Saprotroph | OTU82 | Ascomycota | Unclassified Hyaloscyphaceae | Saprotroph |
|  |  | OTU444 | Ascomycota | *Cadophora* | Endophyte | OTU119 | Mortierellomycota | *Mortierella* | Saprotroph |
|  |  | OTU72 | Ascomycota | *Pezizella* | Ectomycorrhizal- Saprotroph | OTU6 | Ascomycota | Unclassified Helotiales | - |
|  |  | OTU1571 | Mortierellomycota | Unclassified Mortierellomycota | - | OTU88 | Basidiomycota | *Russula* | Ectomycorrhizal |
|  |  | OTU2327 | Ascomycota | Unclassified Ascomycota | - | OTU899 | Ascomycota | Unclassified Hyaloscyphaceae | Saprotroph |
|  |  | OTU2067 | Basidiomycota | *Tomentella* | Ectomycorrhizal | OTU2353 | Basidiomycota | Unclassified Microbotryomycetes | - |
|  |  | OTU1925 | Mortierellomycota | *Mortierella* | Saprotroph | OTU3032 | Unclassified Fungi | Unclassified Fungi | - |
|  |  | OTU1818 | Ascomycota | Unclassified Helotiales | - | OTU2438 | Ascomycota | *Acephala* | Ectomycorrhizal |
| Bacteria | Network hub | OTU3401 | Patescibacteria | Norank WWH38 |  | OTU2780 | Planctomycetota | Norank OM190 |  |
|  |  | OTU2775 | Gemmatimonadota | Norank Gemmatimonadaceae |  | OTU356 | Unclassified Bacteria | Unclassified Bacteria |  |
|  | module hub | OTU1299 | Acidobacteriota | Bryobacter |  |  |  |  |  |
|  | connector | OTU898 | Actinobacteriota | Norank Gaiellales |  | OTU860 | Bacteroidota | Norank BSV26 |  |
|  |  | OTU874 | Myxococcota | *Haliangium* |  | OTU5478 | Actinobacteriota | Unclassified Acidimicrobiia |  |
|  |  | OTU3394 | Bacteroidota | Norank BSV26 |  | OTU470 | Actinobacteriota | *Solirubrobacter* |  |
|  |  | OTU5215 | Actinobacteriota | Norank Acidimicrobiia |  | OTU6575 | Proteobacteria | Norank TRA3-20 |  |
|  |  | OTU3251 | Bacteroidota | Norank S15-21 |  | OTU6174 | Chloroflexi | Norank AD3 |  |
|  |  | OTU5594 | Patescibacteria | Norank Saccharimonadales |  | OTU3286 | Bacteroidota | Unclassified Chitinophagales |  |
|  |  | OTU1454 | Verrucomicrobiota | *Candidatus Xiphinematobacter* |  | OTU5779 | Actinobacteriota | *Conexibacter* |  |
|  |  | OTU48 | Bacteroidota | Norank Kapabacteriales |  |  |  |  |  |
| Bacteria- Fungi | Network hub | OTU3401 | Patescibacteria | Norank WWH38 |  | OTU2780 | Planctomycetota | Norank OM190 |  |
|  |  | OTU2775 | Gemmatimonadota | Norank Gemmatimonadaceae |  | OTU356 | Unclassified Bacteria | Unclassified Bacteria |  |
|  | connector | OTU898 | Actinobacteriota | Norank Gaiellales |  | OTU3554 | Gemmatimonadota | Norank Gemmatimonadaceae |  |
|  |  | OTU3386 | Patescibacteria | Unclassified Saccharimonadales |  | OTU5215 | Actinobacteriota | Norank Acidimicrobiia |  |
|  |  | OTU874 | Myxococcota | *Haliangium* |  | OTU3251 | Bacteroidota | Norank S15-21 |  |
|  |  | OTU3394 | Bacteroidota | Norank BSV26 |  | OTU5594 | Patescibacteria | Norank Saccharimonadales |  |
|  |  | OTU48 | Bacteroidota | Norank Kapabacteriales |  | OTU6575 | Proteobacteria | Norank TRA3-20 |  |
|  |  | OTU860 | Bacteroidota | Norank BSV26 |  | OTU6174 | Chloroflexi | Norank AD3 |  |
|  |  | OTU5478 | Actinobacteriota | Unclassified Acidimicrobiia |  | OTU3286 | Bacteroidota | Unclassified Chitinophagales |  |
|  |  | OTU470 | Actinobacteriota | *Solirubrobacter* |  | OTU5779 | Actinobacteriota | *Conexibacter* |  |

**Table S3** Soil fungal and bacterial network properties in forested wetlands at vegetation type-level.

|  | Network features | Fungi | | | | Bacteria | | | |
| --- | --- | --- | --- | --- | --- | --- | --- | --- | --- |
|  |  | BLW | ASW | BOW | CSW | BLW | ASW | BOW | CSW |
| **Empirical networks** | Total nodes | 89 | 106 | 127 | 95 | 465 | 747 | 639 | 858 |
|  | Total links | 202 | 307 | 255 | 274 | 1138 | 2793 | 1166 | 4940 |
|  | R^2^ of power-law | 0.821 | 0.776 | 0.865 | 0.885 | 0.881 | 0.921 | 0.915 | 0.670 |
|  | Average degree (avgK) | 4.539 | 5.792 | 4.016 | 5.768 | 4.895 | 7.478 | 3.649 | 11.515 |
|  | Average path distance (GD) | 3.550 | 3.096 | 3.762 | 3.339 | 5.246 | 5.175 | 5.972 | 4.330 |
|  | Average clustering coefficient (avgCC) | 0.178 | 0.144 | 0.131 | 0.076 | 0.107 | 0.122 | 0.136 | 0.096 |
|  | Modularity | 0.513 | 0.460 | 0.554 | 0.365 | 0.697 | 0.561 | 0.727 | 0.445 |
| **Random networks** | Average path distance (GD) | 3.012 ±0.065 | 2.817 ±0.051 | 3.450 ±0.073 | 2.793 ±0.058 | 3.598 ±0.039 | 3.382 ±0.023 | 4.189 ±0.051 | 3.126 ±0.018 |
|  | Average clustering coefficient (avgCC) | 0.090 ±0.017 | 0.106 ±0.017 | 0.045 ±0.009 | 0.151 ±0.019 | 0.045 ±0.005 | 0.054 ±0.005 | 0.020 ±0.004 | 0.097 ±0.005 |
|  | Modularity | 0.392 ±0.012 | 0.327 ±0.009 | 0.459 ±0.010 | 0.308 ±0.008 | 0.416 ±0.004 | 0.300 ±0.004 | 0.535 ±0.005 | 0.211 ±0.003 |

**Table S4** Network hubs, module hubs and connectors in the networks within fungal communities at vegetation type-level.

|  |  | OTU | Abundance (%) | Phylum | Genus | Funguild | OTU | Abundance (%) | Phylum | Genus | Funguild |
| --- | --- | --- | --- | --- | --- | --- | --- | --- | --- | --- | --- |
| BLW | module hubs | OTU788 | 0.05 | Basidiomycota | *Tomentella* | Ectomycorrhizal | OTU2059 | 0.03 | Ascomycota | *Lachnum* | Saprotroph |
|  | connectors | OTU2121 | 0.01 | Ascomycota | *Halokirschsteiniothelia* | — | OTU899 | 0.04 | Ascomycota | Unclassified Hyaloscyphaceae | Saprotroph |
| ASW | network hubs | OTU2151 | 0.05 | Basidiomycota | *Lepista* | Saprotroph | OTU356 | 0.54 | Basidiomycota | *Geminibasidium* | Saprotroph |
|  | module hubs | OTU2155 | 0.01 | Basidiomycota | *Cortinarius* | Ectomycorrhizal | OTU2603 | 0.03 | Ascomycota | *Cistella* | Saprotroph |
|  | connectors | OTU2153 | 0.003 | Ascomycota | Unclassified Hyaloscyphaceae | Saprotroph | OTU1011 | 0.003 | Basidiomycota | *Cutaneotrichosporon* | Pathogen |
|  |  | OTU2016 | 0.06 | Ascomycota | *Humicolopsis Pezizomycotina fam Incertae sedis* | — | OTU3192 | 0.01 | Ascomycota | *Trichoderma* | Saprotroph |
| BOW | module hubs | OTU3232 | 0.002 | Basidiomycota | *Sistotrema* | Ectomycorrhizal- Saprotroph | OTU2238 | 0.01 | Basidiomycota | *Hypholoma* | Saprotroph |
|  |  | OTU2339 | 0.02 | Ascomycota | Unclassified Helotiales | — |  |  |  |  |  |
|  | connectors | OTU936 | 0.21 | Ascomycota | *Neonectria* | Pathogen | OTU287 | 0.06 | Basidiomycota | *Hebeloma* | Ectomycorrhizal |
|  |  | OTU1411 | 0.12 | Basidiomycota | *Naucoria* | Ectomycorrhizal | OTU2067 | 0.94 | Basidiomycota | *Tomentella* | Ectomycorrhizal |
|  |  | OTU788 | 0.05 | Basidiomycota | *Tomentella* | Ectomycorrhizal | OTU3107 | 0.50 | Basidiomycota | *Thelephora* | Ectomycorrhizal |
|  |  | OTU2469 | 0.56 | Basidiomycota | *Tomentella* | Ectomycorrhizal | OTU2113 | 0.05 | Ascomycota | Unclassified Helotiales | — |
|  |  | OTU1322 | 0.54 | Basidiomycota | *Tomentella* | Ectomycorrhizal | OTU3144 | 3.40 | Basidiomycota | *Tomentella* | Ectomycorrhizal |
|  |  | OTU261 | 0.01 | Ascomycota | Unclassified Hyaloscyphaceae | Saprotroph | OTU617 | 0.11 | Ascomycota | *Ilyonectria* | Saprotroph |
|  |  | OTU2170 | 2.82 | Basidiomycota | *Naucoria* | Ectomycorrhizal | OTU1792 | 0.01 | Ascomycota | Unclassified Ascomycota | — |
|  |  | OTU2256 | 0.13 | Ascomycota | *Meliniomyces* | Ectomycorrhizal-Saprotroph |  |  |  |  |  |
| CSW | module hubs | OTU1953 | 0.02 | Ascomycota | unclassified Ascomycota | — |  |  |  |  |  |
|  | connectors | OTU654 | 0.03 | Basidiomycota | *Clavaria* | Saprotroph |  |  |  |  |  |

**Table S5** Network hubs, module hubs and connectors in the networks within bacterial communities at vegetation type-level.

|  |  | OTU | Abundance (%) | Phylum | Genus | OTU | Abundance (%) | Phylum | Genus |
| --- | --- | --- | --- | --- | --- | --- | --- | --- | --- |
| BLW | module hubs | OTU5905 | 0.04 | Acidobacteriota | Norank bacterium | OTU6189 | 0.01 | Patescibacteria | Norank Saccharimonadales |
|  |  | OTU1301 | 0.69 | Chloroflexi | Norank TK10 | OTU132 | 0.03 | Bacteroidota | Norank 37- 13 |
|  |  | OTU1582 | 0.004 | Chloroflexi | JG30a-KF- 32 | OTU399 | 0.05 | Myxococcota | *Pajaroellobacter* |
|  |  | OTU1799 | 0.19 | Nitrospirota | *Nitrospira* | OTU650 | 0.13 | Acidobacteriota | *Occallatibacter* |
|  |  | OTU3554 | 0.26 | Gemmatimonadota | Norank Gemmatimonadaceae | OTU5928 | 0.14 | Proteobacteria | Norank JG36-TzT-191 |
| ASW | module hubs | OTU318 | 0.10 | Proteobacteria | *Acidibacter* | OTU5885 | 0.15 | Proteobacteria | Unclassified Burkholderiales |
|  |  | OTU5569 | 0.10 | Proteobacteria | MND1 | OTU3439 | 0.10 | Proteobacteria | *Bauldia* |
|  |  | OTU3444 | 0.10 | Proteobacteria | Devosia | OTU5975 | 0.16 | Actinobacteriota | *Nakamurella* |
|  |  | OTU5362 | 0.04 | Acidobacteriota | Norank Subgroup 2 | OTU3554 | 0.26 | Gemmatimonadota | Norank Gemmatimonadaceae |
|  |  | OTU2918 | 0.17 | Actinobacteriota | *Mycobacterium* | OTU6268 | 0.03 | Actinobacteriota | *Microlunatus* |
|  |  | OTU3533 | 0.03 | Bacteroidota | Norank NS11-12 marine group | OTU658 | 0.19 | Acidobacteriota | *Candidatus Solibacter* |
|  |  | OTU478 | 0.41 | Acidobacteriota | Norank Acidobacteriales |  |  |  |  |
|  | connectors | OTU3917 | 0.03 | MBNT15 | Norank MBNT15 |  |  |  |  |
| BOW | module hubs | OTU318 | 0.10 | Proteobacteria | *Acidibacter* | OTU1059 | 0.07 | Myxococcota | *Haliangium* |
|  |  | OTU305 | 0.34 | Proteobacteria | Norank A21b | OTU2676 | 0.02 | Chloroflexi | Norank SBR1031 |
|  |  | OTU5374 | 0.05 | Acidobacteriota | *Candidatus Solibacter* | OTU897 | 0.01 | WPS-2 | Norank WPS-2 |
|  |  | OTU3554 | 0.26 | Gemmatimonadota | Norank Gemmatimonadaceae | OTU1776 | 0.11 | Actinobacteriota | Norank Gaiellales |
|  |  | OTU5478 | 0.08 | Actinobacteriota | Unclassified Acidimicrobiia | OTU5834 | 0.03 | Patescibacteria | Unclassified Saccharimonadales |
|  |  | OTU5934 | 0.07 | Actinobacteriota | Norank 67-14 | OTU6017 | 0.02 | Gemmatimonadota | *Gemmatimonas* |
|  | connectors | OTU3259 | 0.01 | Chloroflexi | Norank SBR1031 | OTU3209 | 0.01 | Acidobacteriota | Norank Subgroup 18 |
|  |  | OTU1939 | 0.07 | Proteobacteria | Norank Xanthobacteraceae |  |  |  |  |
| CSW | network hubs | OTU3 | 0.04 | Bacteroidota | *Mucilaginibacter* |  |  |  |  |
|  | module hubs | OTU1299 | 0.25 | Acidobacteriota | *Bryobacter* | OTU403 | 0.12 | Actinobacteriota | Norank Gaiellales |
|  |  | OTU4069 | 0.12 | Acidobacteriota | Norank Subgroup 7 | OTU4180 | 0.17 | Actinobacteriota | Acidothermus |
|  |  | OTU870 | 0.003 | Planctomycetota | Norank Gemmataceae | OTU2983 | 0.33 | Actinobacteriota | Norank IMCC26256 |
|  |  | OTU2808 | 0.003 | Acidobacteriota | Norank Subgroup 7 | OTU1762 | 0.003 | Actinobacteriota | Norank Microtrichales |
|  |  | OTU3554 | 0.26 | Gemmatimonadota | Norank Gemmatimonadaceae | OTU6227 | 0.002 | Actinobacteriota | *Pseudonocardia* |
|  |  | OTU1067 | 0.38 | Actinobacteriota | *Acidothermus* | OTU4864 | 0.015 | Actinobacteriota | Norank 67-14 |
|  |  | OTU6207 | 0.002 | Planctomycetota | AKYG587 | OTU3296 | 0.25 | Acidobacteriota | Norank Subgroup 7 |
|  |  | OTU3354 | 0.58 | Acidobacteriota | Norank Acidobacteriales | OTU6108 | 0.21 | Acidobacteriota | *Candidatus Solibacter* |
|  | connectors | OTU3420 | 0.08 | Proteobacteria | *Rhodanobacter* | OTU5286 | 0.10 | Acidobacteriota | *Candidatus Solibacter* |
|  |  | OTU1809 | 0.02 | Acidobacteriota | Norank Subgroup 2 | OTU2273 | 0.05 | Myxococcota | Norank BIrii41 |
|  |  | OTU6504 | 0.01 | Acidobacteriota | Aridibacter |  |  |  |  |


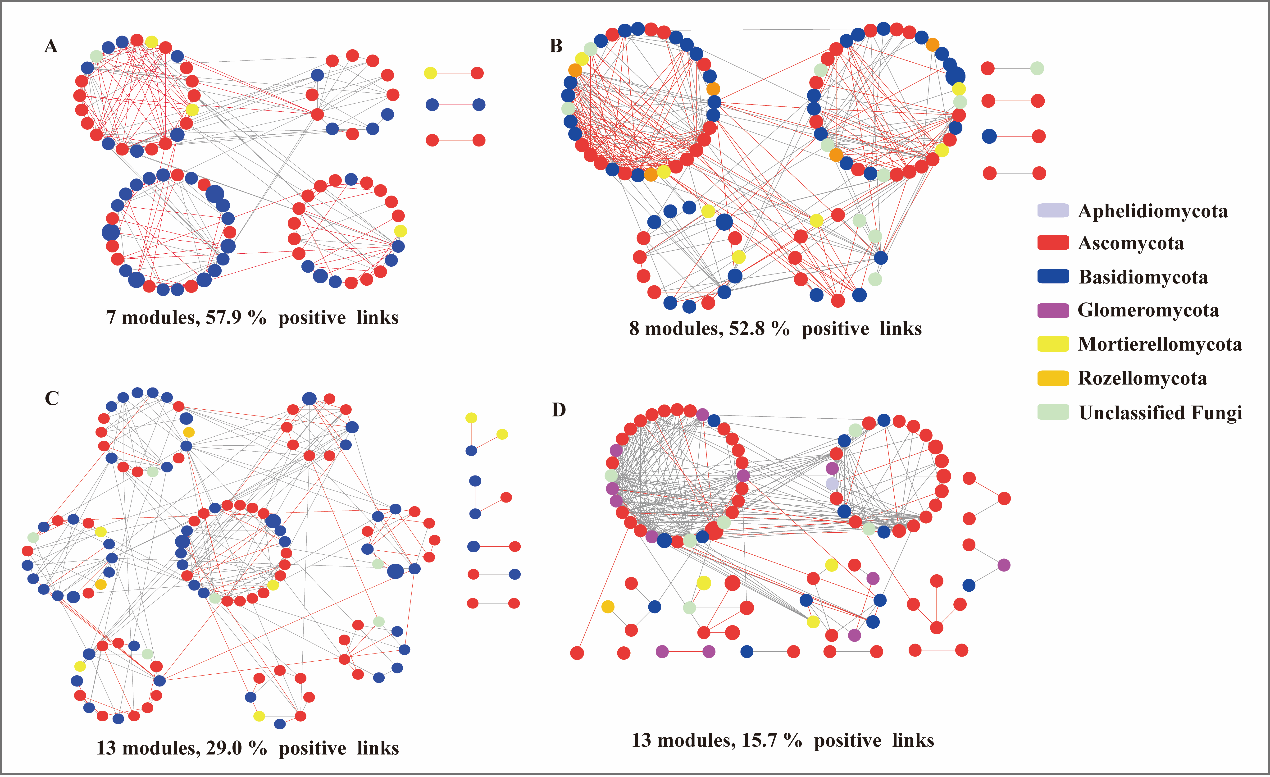


**Fig. S5.** Molecular ecological networks of soil fungal communities at different vegetation types. (A) *B*. *platyphylla* – *L*. *gmelinii* wetland (B) *A*. *sibirica* wetland (C) *B*. *ovalifolia* wetland (D) *C*. *schmidtii* wetland. The identical color of nodes in the network represent the same phylum. Red lines represent positive interactions; grey lines represent negative interactions.


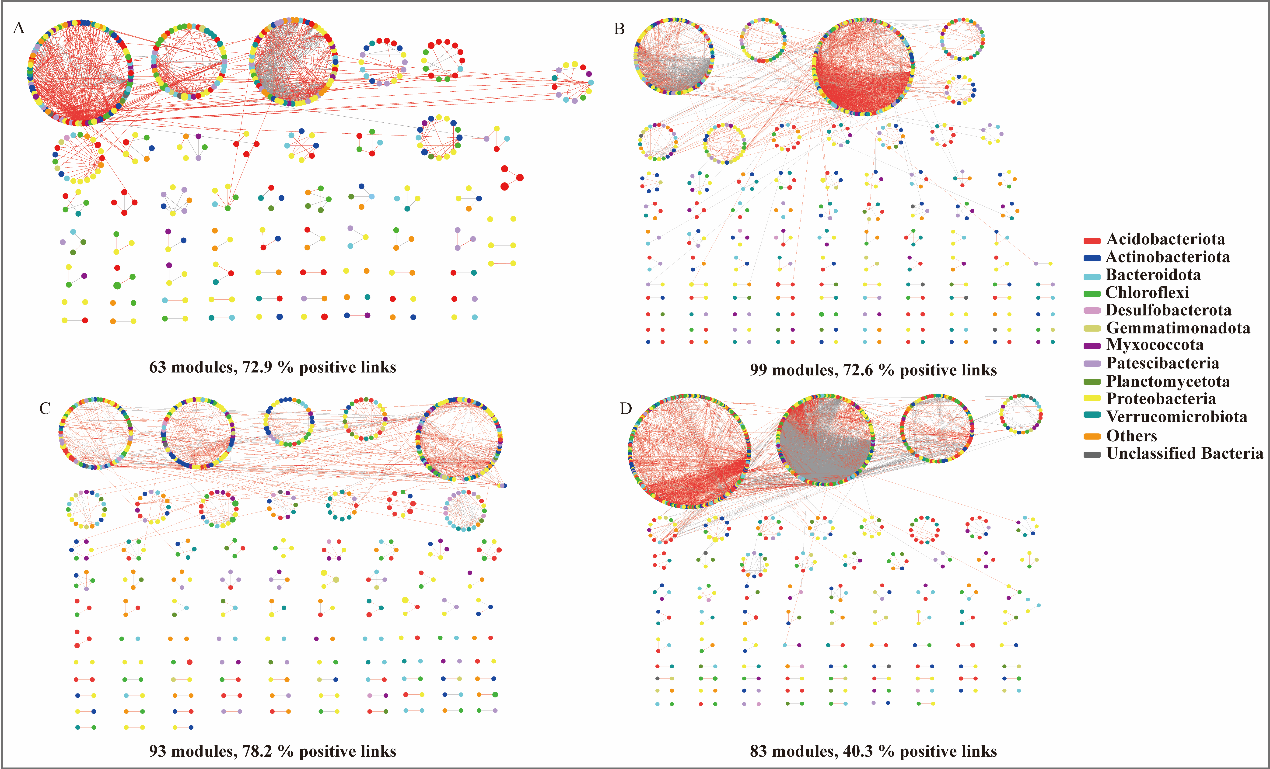


**Fig. S6.** Molecular ecological networks of soil bacterial communities at different vegetation types. (A) *B*. *platyphylla* – *L*. *gmelinii* wetland (B) *A*. *sibirica* wetland (C) *B*. *ovalifolia* wetland (D) *C*. *schmidtii* wetland. The identical color of nodes in the network represent the same phylum. Red lines represent positive interactions; grey lines represent negative interactions.


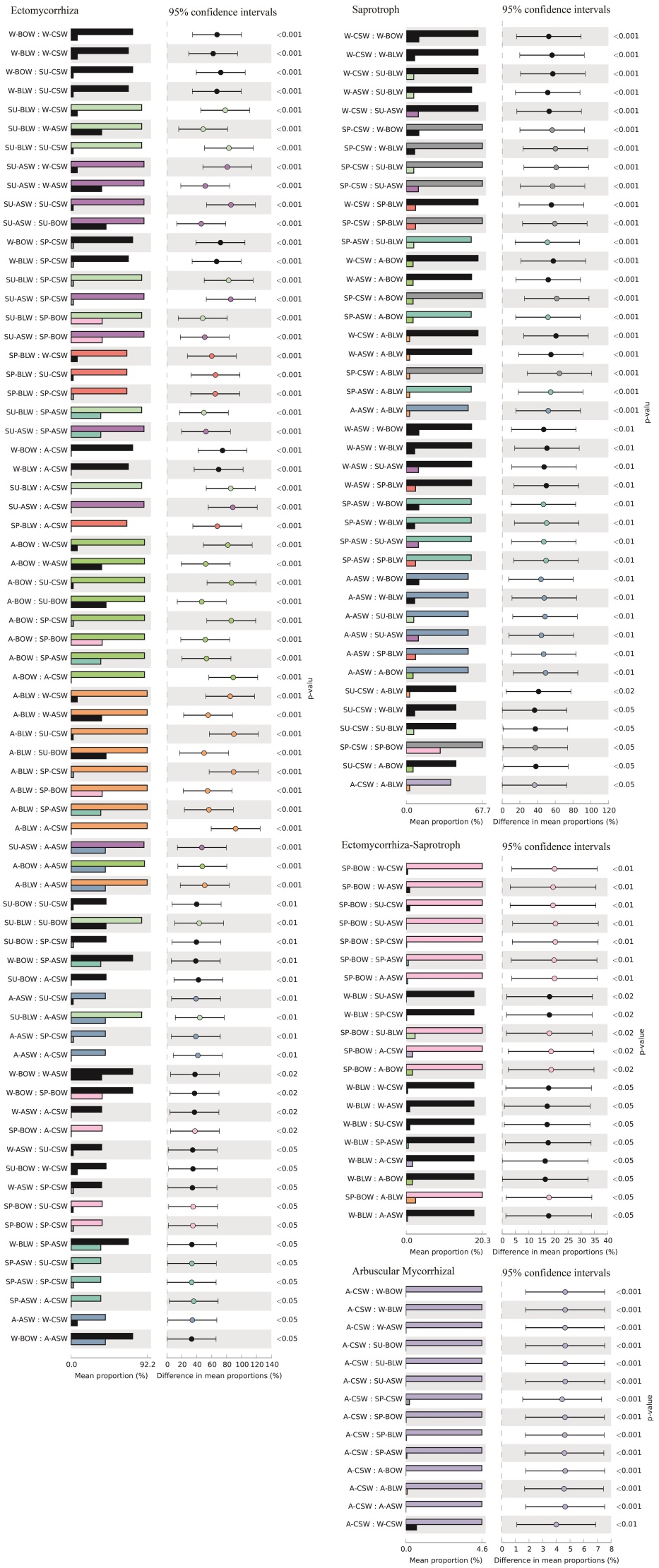


**Fig. S7.** The significance test of the relative abundance of soil total functional fungal community from different wetlands.

**Fig. S8.** Soil nutrient properties in different wetland types.

**DATA AVAILABILITY**

All bacterial and fungal raw sequences have been deposited in the NCBI Sequence Read Archive (No. SRP304662).
